# A deep generative approach to personalized super mario level design

**DOI:** 10.1038/s41598-026-46199-1

**Published:** 2026-04-18

**Authors:** Deniz Baharvand, Nima Saeedi, Sina Samadi Gharehveran, Kimia Shirini

**Affiliations:** 1 Department of Electrical and Computer Engineering, University of Tabriz, Tabriz, Iran; 2 Faculty of Multimedia, Tabriz Islamic Art University, Tabriz, Iran

**Keywords:** Procedural content generation (PCG), Generative adversarial networks (GANs), Deep generative models, Personalized game design, Skill-conditioned level generation, Engineering, Mathematics and computing

## Abstract

Designing game levels that appropriately match a player’s skill level remains a fundamental challenge in procedural content generation, as mismatches in difficulty can lead to player boredom, frustration, and reduced engagement. While deep generative models enable automatic level synthesis at scale, the comparative effectiveness of different GAN architectures for skill-conditioned and personalized level generation remains insufficiently explored. In this work, we investigate the use of generative adversarial networks (GANs) for skill-conditioned procedural content generation by evaluating five architectures: U-Net GAN, StyleGAN, Deep Convolutional GAN (DCGAN), ResNet-GAN, and Spectral Normalization GAN (SN-GAN). Player behavior is clustered into discrete skill groups using Spectral Clustering, and the resulting labels are employed as conditioning signals for level generation. The selected architectures span different convolutional depths, normalization strategies, and regularization mechanisms, enabling a broad and systematic comparison. All models are evaluated using consistent quantitative metrics, including tile distribution entropy, diversity score, discriminator accuracy, generation speed, and pairwise Hamming distance. Quantitative analysis demonstrates that as player skill increases, generated levels exhibit fewer deaths and shorter completion times, while encouraging more complex interactions such as higher jumps and coin collection. Experimental results indicate that ResNet-based and U-Net-style GAN architectures achieve the most favorable balance between level diversity, playability, and training stability, corroborating both qualitative and quantitative assessments. The spectral clustering-based skill-conditioning approach successfully produced personalized levels aligned with each player’s abilities, supporting adaptive procedural content generation across different skill groups.

## Introduction

Designing platform game levels manually is often hard work and time-consuming, particularly for games with a large number of stages such as Super Mario Bros., which feature complex and highly structured level designs^1^. This challenge becomes even more pronounced when design requirements demand unique and customized experiences that adapt to the evolving capabilities of individual players^2^. In such scenarios, there is a growing need for automated systems capable of generating diverse, adaptive, and personalized game content^3,4^.

Procedural Content Generation (PCG) has emerged as a promising solution to this problem^5^. By leveraging algorithmic generation, PCG enables designers to produce large-scale game content with reduced manual effort^6^. However, a central challenge remains: generating content that is not only coherent and playable but also appropriately personalized to a player’s skill level^7,8^. Accurate estimation of player skill is therefore crucial. When the difficulty of a level is significantly lower than a player’s skill, boredom may occur, whereas overly difficult levels can lead to frustration and player dropout^9^. Modeling and predicting player skill within PCG systems allow game content to dynamically adapt difficulty, thereby maintaining player engagement and flow and improving long-term retention.

In this work, player skill levels are inferred by applying Spectral Clustering to gameplay behavior metrics, including locomotion patterns, completion performance, collection behavior, and survival statistics^9,10^.This process results in three distinct skill-based groups: Beginner, Intermediate, and Advanced. These groups are subsequently used as conditioning variables for a set of Generative Adversarial Networks (GANs) to achieve skill-conditioned level generation^11–20^.

Five GAN architectures with diverse architectural and regularization characteristics are explored: U-Net GAN, Deep Convolutional GAN (DCGAN), ResNet GAN, StyleGAN, and Spectral Normalization GAN (SN GAN)^21^. These models differ in convolutional depth, normalization strategy, and conditioning mechanisms, allowing a systematic comparison under consistent experimental settings^22,23^.

All player behavioral features were standardized using Z-score normalization prior to clustering to ensure stable and balanced contribution from each feature. Although Min–Max scaling produced slightly higher Silhouette Scores during preliminary analysis, it introduced instability when cluster labels were used as conditional inputs for GAN training, due to amplified boundary effects from skewed distributions and outliers. This rationale ensures reproducible and stable skill-conditioned generation. All models are trained on level corpora derived from the Super Mario AI Framework and the Video Game Level Corpus (VGLC), which provide standardized tile-based representations and structural constraints that ensure playability while allowing meaningful visual and structural variety.

By conducting a comprehensive comparison of multiple GAN architectures under identical experimental conditions, this study aims to clarify trade-offs between design choices and model families, enabling personalized and skill-adaptive procedural content generation in platform games.

The generative models are evaluated using a consistent set of qualitative and quantitative criteria, including difficulty appropriateness, structural diversity, and computational efficiency^3,4,10,12,24–28^. By conducting a comprehensive comparison of multiple GAN architectures under identical experimental settings, this study aims to clarify the trade-offs between different design choices and model families for enabling personalized and skill-adaptive procedural content generation in complex platform games.

The main contributions of this work can be summarized as follows:

1. We propose a unified skill-conditioned procedural content generation pipeline that integrates spectral clustering of player behavior with conditional GAN-based level synthesis, enabling personalized game level generation.
2. We present a systematic and fair comparison of five GAN architectures—U-Net GAN, DCGAN, ResNet GAN, StyleGAN, and SN GAN—under identical training conditions and evaluation metrics for skill-adaptive Super Mario Bros. level generation.
3. We introduce a comprehensive evaluation framework that jointly analyzes controllability, diversity, difficulty appropriateness, and computational cost, offering practical insights into trade-offs among different generative model families for personalized PCG.

The methodology and experimental setup are detailed in the next section, followed by the presentation and discussion of the results.

## Related work

PCG research has shifted from early rule based and grammar driven PCG systems to pipelines fuelled by machine learning techniques that can learn design characteristics directly from gameplay data in the last twenty years. One of the first such grammar evolution works by Shaker et al. (2011) was based on Super Mario Bros^24^. While they could show that grammars of a pre-defined structure can generate different layouts that respect some design constraints, personalization of such systems is generally low. PCG was later extended by methods such as those by Togelius et al. that use search based PCG methods such as evolutionary algorithms to generate racing tracks, later adapted to platformers for automatic difficulty generation^29^. These optimisation based methods generally generated more diverse layouts, but very few of them modelled player behaviour explicitly, so the new content was not generally tailored to specific skill profiles.

Summerville and Mateas combined sequence modelling with the VGLC to generate stages automatically that both matched human design preferences and kept a high playability score^30^. This behaviour conditioning approach was replicated in other genres, including puzzle design^31^ and first person shooters^21^, with both approaches showing the potential in adapting game content to actual player actions^27^. With the recent explosion in the use of deep learning, GANs have been used to produce PCG by learning latent distributions of structural patterns directly from data rather than via rules encoded by human experts. For example, Volz et al. showed how Deep Convolutional GANs could be applied to Mario level generation^1^. This was done with some additional features such as an automated playability check and a playthrough score based on visual similarity, but the general approach allowed levels to be generated and assessed without human expert designers involved. StyleGAN2 by Karras et al. (2020) further extended the controllability of GANs by style based manipulation of the latent space^32^.These GANs have also been applied in PCG research, but their application to adaptive PCG in general is still at a very early stage. Research in other areas of deep learning has also been used to further broaden the range of PCG methods, including autoencoder based architectures (Jain et al., 2016)^52^, plug and play generative models (Nguyen et al., 2017)^51^, and more recently diffusion based synthesis (Ho et al., 2020)^50^. However, very few such models condition the generation process on player performance in some way. A solution to this problem is given by the cluster driven conditioning of generation, first used by Guzdial et al. (2017) to automatically divide players into skill groups by using gameplay features including total jump count, coin count, death count, and completion time. While many early works including this work used K Means clustering, more recent works have found spectral clustering gives a more distinct separation of player profiles, and that this can lead to more reliable conditioning signals for generative models^30^. Personalised clustering methods have also been applied to generate content in non-platform games, including MMORPG quest generation (Baldwin et al., 2017)^32^ and adaptive tutorials (Anderson et al., 2020).

PCG research has thus increasingly trended towards architectures that not only allow generative capability, but also allow for some adaptation to specific players. The recent exploration of latent variable evolution in GANs (Fontaine et al., 2021)^30^ and RL based content generators (Justesen et al., 2019) is part of this movement towards a PCG framework that includes both goal driven optimisation and high fidelity content generation. The approach used in this work directly models on this research, and models skill level using spectral clustering of gameplay behaviour into three high level skill groups: beginner, intermediate and advanced. Five different GAN architectures are then conditioned on the player skill group, including a ResNetGAN, Deep Convolutional GAN, StyleGAN, Spectral Normalisation GAN, and standard Wasserstein GAN. These are used to try and reliably generate diverse, playable and skill adaptive stages, as both the dataset from the Guzdial framework and the Super Mario AI platform are used for this.Beyond game-focused procedural content generation, generative adversarial networks have demonstrated strong effectiveness across a variety of data-intensive application domains that face similar challenges of data scarcity, high dimensional structure, and the need for conditional synthesis. In remote sensing, frequency-to-spectrum mapping GANs have been proposed for semi-supervised hyperspectral anomaly detection, where adversarial learning enables robust modeling of spectral distributions under limited labeled data^33^. Similarly, GAN-based handwritten text generation has been employed for Tibetan script data augmentation, producing high-fidelity synthetic samples to improve recognition performance in low-resource language settings^24–26,34^. These applications highlight the ability of GAN frameworks to learn complex conditional distributions and generate diverse yet structured outputs across heterogeneous domains. Such properties directly motivate the adoption of GAN-based architectures in this work, where skill-conditioned level synthesis requires both controllability and diversity to support personalized procedural content generation.

## Methodology

The proposed model is a generative approach, built upon a ResNet for the generation of Super Mario Bros levels, conditional on the user skill set^35^. Spectral clustering based on a player’s gameplay metrics (jumps, time taken, coin pickup frequency, and death count) is used to separate users into different player skill levels, with the labels used as conditional inputs to the GAN^11^. This allows for a stage to be synthesized which is unique and more suited to the user’s skill. The ResNet architecture also uses residual connections to allow for easier gradient flow during backpropagation as well as allowing for more feature reuse, resulting in more stable training and higher quality of outputs^36^. The other four baseline models for comparison are the original Deep Convolutional GAN (DCGAN)^36^, the style based latent space generator StyleGAN^37^, the Spectral Normalisation GAN (SN GAN)^37^ and U-Net GAN^38^. Each model is run in an identical environment, with the Super Mario AI Framework being used for a standardized representation of tiles. Metrics used to compare the models quantitatively are Playability Rate, Diversity Score, Personalization Accuracy, Fréchet Inception Distance (FID) and Generation Speed^39–42^.

**Fig. 1:**
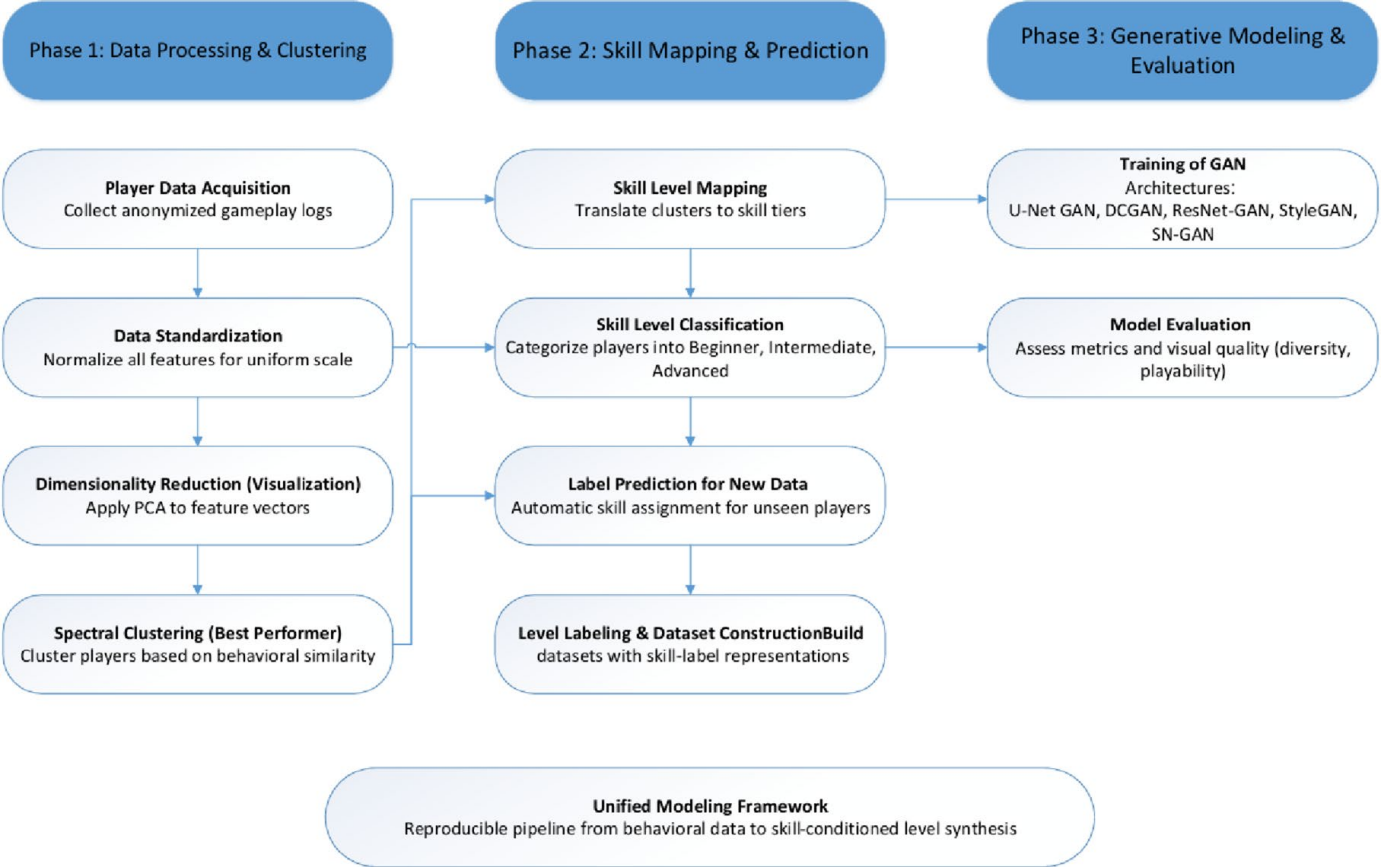
Skill-conditioned procedural level generation workflow illustrating data processing, skill mapping, and GAN-based level synthesis phases.

Figure 1 presented skill-conditioned procedural level generation framework unifies behavioral analytics and generative modeling in three sequential stages. In Stage 1 (Data Processing & Clustering), anonymized player gameplay logs are collected, normalized, and compressed using Principal Component Analysis (PCA) for dimensionality reduction, and subsequently clustered via spectral clustering to identify distinct player behavior profiles.In Stage 2 (Skill Mapping & Prediction), clustered players are assigned to discrete skill levels (Beginner, Intermediate, Advanced), and the same labeling pipeline is applied to unseen input data to construct skill-annotated datasets.Finally, in Stage 3 (Generative Modeling & Evaluation), multiple Generative Adversarial Network (GAN) variants—U-Net GAN, StyleGAN, Spectral Normalization GAN (SN-GAN), Deep Convolutional GAN (DCGAN), and ResNet GAN—are employed to condition procedural level generation on player skill, followed by quantitative and qualitative evaluation of level diversity and quality.Overall, the framework establishes a reproducible skill-conditioned pipeline for procedural content generation driven by player behavioral data.

### Player modeling

Player skill levels were derived from the Guzdial dataset (Guzdial et al., 2017)^32^, which provides detailed gameplay metrics—including number of jumps, coins collected, deaths, and completion time—for 74 players across 11 levels of Super Mario Bros. No synthetic data augmentation was applied; all analyses were conducted on real player data. Player behavior was clustered using the Spectral Clustering algorithm^10^ after evaluating multiple clustering approaches (K-Means, DBSCAN, Agglomerative Clustering, and Gaussian Mixture Models). Spectral Clustering was selected due to its superior Silhouette Score (0.203511) and its ability to capture complex, non-linear boundaries in the behavioral feature space. While an initial four-cluster configuration (Beginner, Intermediate, Advanced, Professional”) was explored, the Professional” cluster showed substantial overlap with the Advanced” group and contained few samples, which reduced statistical robustness. Therefore, the final model adopts a three-cluster solution (Beginner, Intermediate, Advanced”), which provides improved cluster stability and clearer separability in player behavior. These skill clusters were subsequently used as conditioning inputs to the GAN models for personalized level generation.

### Data collection and description

To train and evaluate the generative models, this study utilizes three primary datasets, each serving a distinct purpose in the research pipeline:

#### Guzdial player behavior dataset

To train and test the generative models, this research makes use of three datasets. Each dataset is used at a different stage of the research pipeline. The Guzdial data set consists of fine‑grained play‑through logs of 74 players across 11 Super Mario Bros. levels^32^. These behavioral events include game‑specific actions (jumps, coin collections, deaths), time‑elapsed, and more. The fine‑grained, behavioral features are used as the player model, from which player skill can be inferred.

Raw play‑through logs were compiled as a dataset with both event‑based and rate‑based descriptors (jumps_per_sec, coins_per_sec, deaths_per_sec, session_duration, time_to_first_coin), as well as aggregate event counts (JumpStart, DieByGreenKoopa, WonLevel, etc.). Feature vectors for each player were concatenated for all levels and used directly for clustering; no synthetic augmentation was performed. Players were grouped by Spectral Clustering, and segmented into three skill levels: Beginner, Intermediate, and Advanced. The inferred skill labels are used as the conditional input for the GAN‑based level generation models. The public Guzdial repository (https://github.com/guzdialg/MarioData) includes all CSV logs, corresponding level images, and survey responses for a multidimensional overview of the player–game interaction. Table 1 is a sample of recorded gameplay events such as movement, coin collection, and deaths across multiple levels.

**Table 1:** Sample event log from the Guzdial dataset.

Index	Event	Time (s)	Player ID	Level type
0	LittleStateStart	0	5,564,920	TestLevel2
1	StartLevel	0	5,564,920	TestLevel2
2	RightMoveStart	73	5,564,920	TestLevel2
3	BlockCoinDestroy	108	5,564,920	TestLevel2
4	BlockCoinDestroy	182	5,564,920	TestLevel2

####  Video game level corpus (VGLC)

The Video Game Level Corpus (VGLC) is a collection of semantically-rich representations of original Super Mario Bros levels that are formatted as 16 × 128 tile grids. Each cell in the grid represents an in-game element including X to represent a ground block, </> for pipes, E for enemies, o for coins, and multiple platform or background tile types^43^. In our experiments we programmatically filtered all available Super Mario Bros. level files (.txt files) from VGLC for levels that are valid both in terms of containing a platform and interactive tiles. This left us with a corpus of semantically rich and playable levels that were then extracted into arrays of tile indexes. In the array extraction each level is parsed based on a single tile dictionary that maps tile semantics for the entire corpus, and each input was either padded or cropped to have the fixed resolution of 16 × 128. The arrays of tile indexes became the source set of levels used for training the GAN based generative models. In this way the models learned the distributions of spatial and semantic features of real Nintendo designed levels to produce levels that would be valid for gameplay and conditioned on the clustered player groups from Guzdial dataset^30^.

#### Super Mario AI framework dataset

The Super Mario AI Framework is a standard platform to evaluate levels and test them with agent based playtesting. It has been used in many different research papers related to PCG and is a standard platform in PCG Challenges. The data set associated with the framework has verified Super Mario Bros. level files compatible with AI agents, including rule based agents and search based agents (for example an A* pathfinding agent). It also contains logs in CSV format of the gameplay, images of the level mapped to tiles, and a summary of the agent performance. These can all be used to ensure the playability and completeness of generated levels, in terms of game functionality, game structure, and in-game navigability, rather than purely visual and content-based similarity.

The main usage of the framework in our research is to calculate the playability metrics and verify the structure of the generated levels. As the process for traversing the graph with BFS, defining passable tiles, and the metrics calculations are similar to the methods used in the Super Mario AI Framework, these are detailed in “Discussion and results” (Evaluation Metrics). The dataset is combined with the Guzdial Player Behavior Dataset and VGLC structural dataset to form the basis of our pipeline for quality verified, personalized level generation.

### Data preprocessing

Both the tile-based raw level data from the VGLC dataset and the player behavior cluster labels acquired from Spectral Clustering (“Spectral clustering”) were preprocessed for uniformity and neural-network compatibility prior to training the generative models.

#### Level preprocessing

Individual Mario level files were tokenized into a fixed length array of shape 16 × 128 with each value representing a tile type. Levels of any other size were padded with fill/noise (or trimmed, in the case of overflow) so that all data have the same shape.

A project specific tile_dict mapping was also created, automatically generated from all parsed levels, ensuring total consistency between preprocessing and model training. An example of the full mapping is shown in Table 2. This dictionary is identical to the one stored in mario_tile_dict.npy upon creation of the dataset^43^. Levels were then translated from character arrays to integer arrays with this mapping and the resulting arrays were saved in .npy format for downstream training and analysis. The correspondence between tile indices and symbolic characters used during preprocessing is shown in Table 2.

**Table 2:**
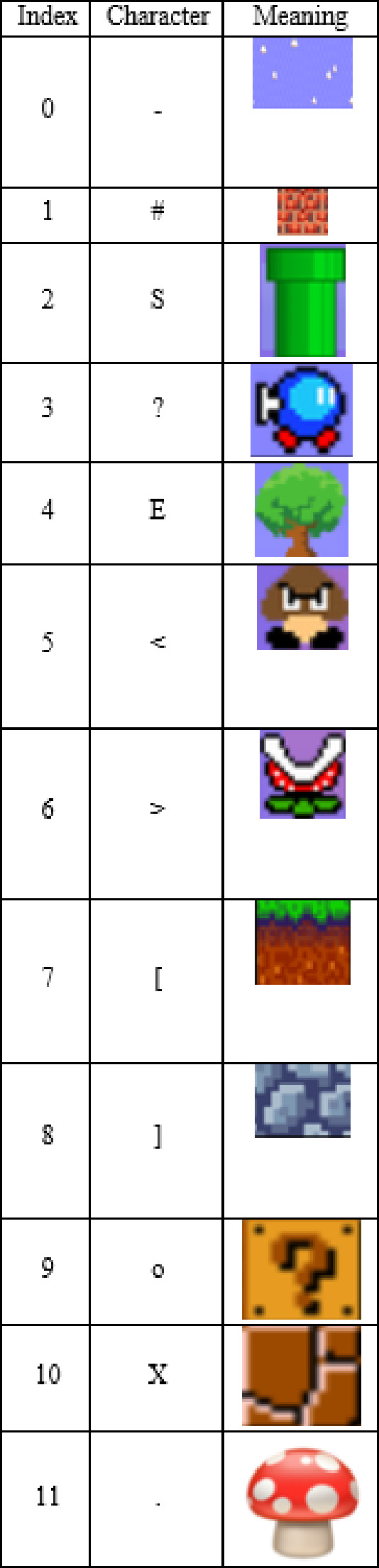
Tile-to-symbol mapping used for Mario level preprocessing and reconstruction.

The GAN models directly generate tile-based levels in a raw array format, where each cell denotes an abstract tile index. These outputs were then converted into text-based level files using the *VGLC* representation, mapping numeric indices to symbolic characters shown in Table 2. Finally, semantic sprites corresponding to each character were visually re-inserted to reconstruct playable Mario levels from the generated text layouts.

#### Normalization for GAN training

Since GAN models like DCGAN have the tangent activation function (Tanh) at the output layer of the generator, the mapping of the values to the output range of this function (that is [− 1, 1]) is required^44^. For this reason, the numerical values of each element (which were in range of [0, 12]) were normalized by using a standard linear transformation (min–max normalization). The equation for this transformation is written in Eq. (1).

1 $$value\,normalized=2 \times \frac{{x - \hbox{min} \,x}}{{\hbox{max} \,x - \hbox{min} \,x}} - 1$$

The variable x corresponds to the principal numeric value of the item, having min_x = 0 as its minimum (i.e., the smallest value of the initial interval) and max_x = 12 as its maximum (i.e., the largest value of the initial interval). The normalized value value_normalized is defined in the interval [− 1,1]. This normalization not only makes the model outputs consistent with the tangent range, but also contributes greatly to the stability and improvement of the training process of GAN-based models.

#### Feature reduction and visualization

Dimensionality reduction is applied to the feature vectors used for clustering as a preprocessing step to facilitate intuitive visualization of player behavior and to examine the separability of player types. The numeric feature vectors are first standardized to zero mean and unit variance using z-score normalization with StandardScaler^35^, ensuring that all features contribute equally to the distance metrics used during clustering. PCA is then employed to project the standardized feature vectors into a two-dimensional subspace via an orthogonal linear transformation while preserving the maximum total variance^36^. This reduced representation provides a compact visualization of player clusters and an interpretable overview of cluster separability across different clustering algorithms, including Spectral Clustering, K-Means, Agglomerative Clustering, DBSCAN, and Gaussian Mixture Models. Identical color maps and marker sizes are used across all visualizations to reduce cognitive bias and to facilitate direct comparison when evaluating cluster separability.

##### Principal component analysis (PCA)

Principal Component Analysis (PCA) is a linear dimensionality reduction algorithm which projects high dimensional data to low dimensional space through finding orthogonal principal components that maximise variance. The behaviour of our players were described by a high-dimensional space with over 100 potentially correlated features such as completion times, jump counts and deaths, which made it difficult to visualise or cluster the data directly. To enable a clear two-dimensional visualisation of the data to compare all the clustering algorithms we tested as well as our top performer Spectral Clustering, we ran PCA on our normalised feature matrix, reducing it to two components. We used the StandardScaler method to standardize all of the features before performing PCA so that all of the metrics have equal weight, zero mean, and unit variance^45^. The implementation used in our project was sklearn. Decomposition PCA(ncomponents = 2), which resulted in two PCs, PC1 and PC2, that contain the global variance structure in the dataset. In other words, we are mathematically defining this as follows: (“Related work”).

2 $${X_{PCA}}=PCA(ncomponents=2)\left[ {{X_{scaled}}} \right]$$

where Xscaled​ denotes the standardized matrix of dimensions m×N (players × features) and ​ $\:{\mathrm{X}}_{\mathrm{P}\mathrm{C}\mathrm{A}}\:$represents the reduced two-dimensional matrix. Preserving the global variance pattern means that PCA offers an objective way to evaluate the compactness and separation of clusters in the reduced space. This is in contrast to e.g. t SNE, which optimizes local neighbourhood structure.

### Prediction of player skill level

Player skill-based content personalization was achieved by clustering players into three skill-based groups using unsupervised learning. The most important behavioral features obtained from the Guzdial dataset include temporal features, interaction features, risk-taking, and proficiency features. The clustering process was preceded by feature normalization using StandardScaler, which standardizes features to have zero mean and unit variance. This normalization ensured that each behavioral feature contributed equally to the clustering process. Although Min–Max scaling resulted in a slightly higher Silhouette Score during preliminary clustering experiments, it introduced instability when the resulting cluster labels were used as conditional inputs for GAN training. Due to skewed behavioral feature distributions and the presence of outliers, Min–Max normalization amplified boundary effects, leading to unstable conditional signals and oscillatory GAN training behavior. Therefore, z-score standardization was adopted to preserve relative feature variance and ensure better compatibility with conditional GAN models. Several clustering algorithms were evaluated, and Spectral Clustering was selected as the most suitable method based on clustering quality and separability. Spectral Clustering was ultimately used to divide players into beginner, intermediate, and advanced skill groups. These three skill-based groups were then employed as conditional labels for training the GAN models to generate adaptive, skill-conditioned game levels.

Evaluation metrics:

To comprehensively evaluate the quality, diversity, and adherence of the generated outputs to the target skill level, the following quantitative metrics are computed:

1. Playability rate (PR)

This metric is calculated using a Simulated Automated Agent equipped with a basic movement strategy (move right, basic jump logic).

3 $$PR=\frac{{{N_{Completed}}}}{{{N_{Total}}}} \times 100$$

where

- ${N_{Total}}$: The total number of levels generated by the model for a specific skill condition.
- ${N_{Completed}}$: The number of levels successfully traversed (at least 90% of the level length) by the automated agent without failing.

2. Diversity score (DS)

To quantify the diversity in level tile composition, the inverse of the Shannon Entropy of the tile distribution is used. Assuming K distinct tile types (e.g., ground, enemy, coin, obstacle) exist in the dataset.

4 $$DS= - \frac{1}{{\sum\limits_{{K=1}}^{K} {{p_i}\log ({p_k})} }}$$

where

- ${p_k}$​: The frequency ratio of tile type k in the generated level relative to the total number of tiles in that level.
- Note: If ${p_k}=0$, then${p_i}\log ({p_k})=o$.

#### K-means clustering

K-Means is one of the most commonly used clustering algorithm^46^. K-Means Clustering is a partition based clustering method which attempts to group a set of n observations into k clusters such that the sum of the squared Euclidean distance of each observation to the centroid of its cluster is minimized. This model is used especially for identifying the most tightly packed clusters that are of spherical or ball-shaped and with variance (size) similar to each other. K-Means’ primary disadvantages in general are that it is sensitive to noise and outliers and performs poorly when dealing with clusters that have non-globular shapes. This is a significant problem in player statistics since it is real-world data and is very high dimensional and has a complex manifold structure. As such, it was used in the first phase of experimentation for completeness, but Spectral Clustering was eventually used instead.

#### DBSCAN (density-based spatial clustering of applications with noise)

DBSCAN is a density-based, non-parametric clustering algorithm that forms clusters by discovering areas of high data-point density and identifying points in sparse areas as noise^47,48^. The main benefit of this approach is that it does not require a-priori knowledge of the cluster count and can yield stable results on datasets with non-linear, complex structure. The latter characteristic makes DBSCAN a particularly attractive choice for clustering of the generated player feature space. The key drawback is that performance is extremely sensitive to the choice of the hyper-parameter settings, as the two main parameters, ε and minPts, that determine the nature of the neighborhoods used for generating the clusters have to be carefully selected, as even a small change can lead to a dramatically different result. As the feature space of the extracted players was both high-dimensional and contained uneven density distribution, it was difficult to get a globally optimal and stable solution that was robust to the hyper-parameter settings. As such, while DBSCAN was experimented with, its performance did not compare with Spectral Clustering on any of the considered metrics (Silhouette and Davies-Bouldin in particular), and as such it is not used in the final model.

#### Agglomerative hierarchical clustering

Agglomerative Hierarchical Clustering is a top-down approach^49^. The algorithm starts by assigning every observation to its own cluster. Then, each pair of clusters is merged (successively), where the pair of clusters that maximizes some similarity measure (e.g. Euclidean distance) according to some linkage criteria (e.g. Ward’s, average, complete linkage) is selected to be merged, until the termination condition is met. The main advantage of this approach is that it can identify (visually) the hierarchical structure of the data, through a dendrogram, which can highlight different levels of sub-structures or nested relationships of the players’ skill profiles. However, this approach can be computationally very inefficient on large scale problems, and the number of clusters has to be selected manually by cutting the dendrogram, which can be subjective. In the feature space at hand, Agglomerative Clustering achieved lower cluster separation (performed worse with Silhouette and DBI) compared to Spectral Clustering, thus it was not used for the final prediction.

#### Spectral clustering

Spectral Clustering is a graph‑based clustering algorithm which can identify complex, non‑linear, arbitrarily shaped clusters^43^. It views clustering as a graph partitioning problem by representing data instances as nodes on a graph connected by weighted edges representing pairwise similarity between instances. The Graph Laplacian is calculated and data is projected into a lower‑dimensional embedding space by using the eigenvectors of the Graph Laplacian corresponding to its smallest eigenvalues, on which the data becomes linearly separable. K‑Means is then performed on the lower‑dimensional data to find cluster assignments. Spectral Clustering was ultimately chosen for the player skill classifier due to its demonstrated ability to capture the manifold structure of the high‑dimensional player behavior features and its highest Silhouette Score (0.203511).

#### GMM (Gaussian Mixture Model)

The Gaussian Mixture Model (GMM) takes a probabilistic approach^50^. It assumes that the data is best described by a superposition (mixture) of a finite number of Gaussian distributions. Instead of assigning each data point to exactly one cluster, as in hard-assignment methods like K-Means^46^, GMM defines a cluster as a single Gaussian distribution with its own mean, covariance and mixing coefficient, with data points probabilistically assigned to each. Expectation Maximization (EM) can be used to estimate the parameters of these distributions. The GMM is much more flexible than distance-based methods. Clusters can have different sizes, different shapes (defined by the covariance matrix) and different densities, and the model can easily handle overlapping clusters. Despite this, and its clear advantages from a statistical modeling perspective, GMM on the standardized player feature set failed to produce the desired results of cleanly separated clusters. Internal validation scores (Silhouette Score of 0.116679, for example) were much worse than what was achieved by the graph-based method. It was thus demoted to an interesting alternative and comparison, with Spectral Clustering being chosen for the final player skill prediction task^51^.

### Comparative evaluation of normalization type methods

**Table 3:** Comparison of clustering algorithms using different normalization methods and internal validity indices The best-performing values for each evaluation metric are highlighted in bold .

Model	Normalization type	Silhouette score	Davies–Bouldin Index (DBI)	Calinski–Harabasz Index (CHI)
K-Means	Standardization	0.152000	**1.562700**	13.460000
DBSCAN	Standardization	− 0.115100	2.206200	2.330000
Agglomerative	Standardization	0.171000	1.569800	17.310000
Spectral	Standardization	**0.210400**	1.686700	12.620000
GMM	Standardization	0.152000	**1.562700**	13.460000
K-Means	Min–Max Scaling	0.1146	1.8627	13.8695
DBSCAN	Min–Max Scaling	− 0.1151	2.2062	2.3315
Agglomerative	Min–Max Scaling	0.1710	1.5698	**17.3133**
Spectral	Min–Max Scaling	0.2035	1.5875	12.0756
GMM	Min–Max Scaling	0.1146	1.8627	13.8695

In Table 3, we present the internal clustering metrics for the five candidate algorithms—K Means, DBSCAN, Agglomerative Clustering, Spectral Clustering, and Gaussian Mixture Models (GMM)—using two data normalization approaches (Standardization, Min–Max Scaling) along with three internal evaluation metrics—Silhouette Score (Eq. 1), Davies–Bouldin Index (DBI) (Eq. 2), and Calinski–Harabasz Index (CHI) (Eq. 8)—to measure cluster quality. Higher Silhouette Score values indicate more separated inter clusters and cohesive intra clusters, lower DBI values indicate more compact clusters, and higher CHI values indicate more between cluster dispersion versus within cluster variance. The table shows that Spectral Clustering with Standardization yielded the best silhouette score of 0.210400, K Means and GMM with Standardization produced the lowest DBI of 1.562700 (closely followed by Spectral Clustering with Standardization with a DBI of 1.686700), and Agglomerative Clustering with Min–Max Scaling obtained the largest CHI of 17.313300, however, at the expense of the silhouette score (0.171000). Since no single configuration produces the best results across all three metrics, a trade off was required. As the downstream goal of the study is to create stable and reproducible skill-level labels for the GAN-based level generation pipeline, the Silhouette Score was given the highest priority as it measures cluster separability, while a lower priority was assigned to DBI and CHI provided that they did not exhibit outlier (poor) values. In the end, Spectral Clustering with Standardization was chosen as the final clustering method for the GAN-based level generation pipeline as it has the best Silhouette Score (0.210400), an acceptable DBI value of 1.686700, and a relatively high CHI value of 12.620000, consistently producing three stable clusters (Beginner, Intermediate, and Advanced) across multiple runs.

### Procedural content generation models

**Fig. 2:**
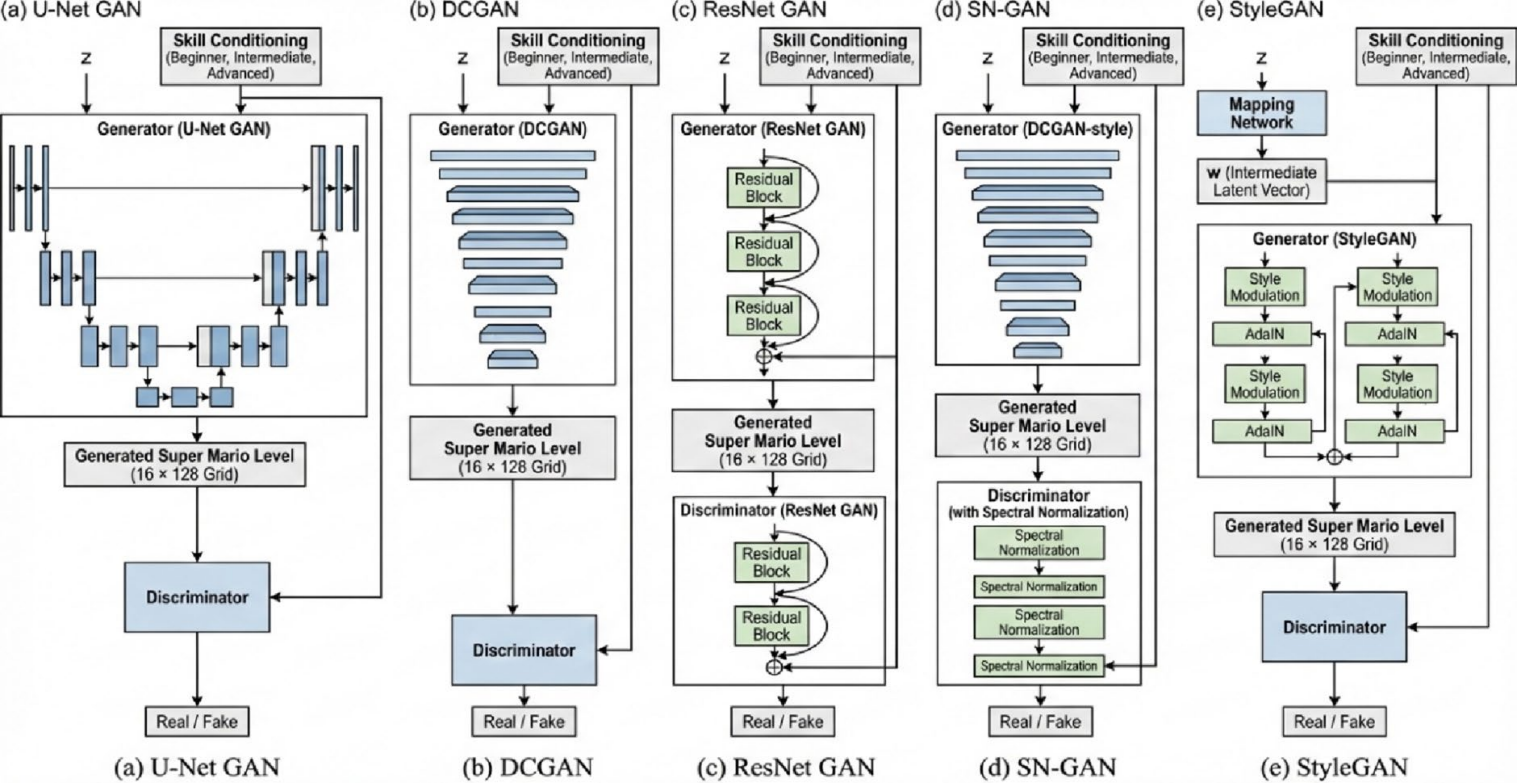
Schematic architectural comparison of the five GAN models evaluated for skill-conditioned Super Mario level generation. (**a**) U-Net GAN, (**b**) DCGAN, (**c**) ResNet GAN, (**d**) Spectral Normalization GAN (SN-GAN), and (**e**) StyleGAN.The figure highlights the main generator–discriminator structures and conditioning mechanisms of each model. In all cases, player skill labels (Beginner, Intermediate, Advanced) are used as conditional inputs, and the generator produces a 16 × 128 tile-based Super Mario level evaluated by the corresponding discriminator.

To generate personalized Super Mario levels, we evaluated and compared several generative models.In summary, five GAN architectures were implemented and evaluated under identical training and evaluation settings: U-Net GAN, DCGAN, ResNet GAN, Spectral Normalization GAN (SN-GAN), and StyleGAN. Detailed architectural descriptions are provided in “UNet GAN”, “DCGAN (Deep Convolutional GAN)”, “ResNet GAN”, “SN-GAN (Spectral Normalization GAN)”, “StyleGAN (Style-Based Generative Adversarial Network)”, ensuring consistency across all analyses. As illustrated in Fig. 2, the five GAN architectures differ in their generator–discriminator designs and conditioning mechanisms.

#### UNet GAN

UNet GAN embeds the U Net architecture in the generator of a Generative Adversarial Network. U Net is a convolutional network designed for image segmentation tasks^22,44,52^. It has a symmetric Encoder–Decoder structure in which skip connections feed the output of each encoder layer to the input of the corresponding decoder layer. They transfer high resolution feature maps from the contracting path to the expansive path, bypassing the feature down sampling that would otherwise cause spatial information loss. The encoder path of U Net can be viewed as extracting an abstracted representation of the input, and the decoder path reconstructing the output given this representation, aided by spatial information from the skip connections. This architecture adapted to the generation of grid based Super Mario levels allows the generator to leverage both the local spatial detail needed to accurately maintain specific local tile configurations which are often required for gameplay (no tile can be placed arbitrarily), and to generate high quality detail where the exact position of each tile in the output can be highly impactful on the game. The adversarial objective supports this detailed spatial requirement with a bias toward well structured, stylistically varied outputs in the manner of those seen in the source dataset. These properties make UNet GAN a promising architecture for generation of high quality, complex, grid based spaces with fine grained spatial control.

The optimization of UNet GAN is exactly that of a vanilla GAN, now conditioned. The objective for the vanilla generator G is to get the discriminator D to classify samples drawn from G as real (Eq. 5).

5 $$\mathop \mathrm{L}\nolimits_{G} = - {{\mathbb{E}}_{z\sim {p_z}}}\left[ {\log D\left( {G\left( z \right)} \right)} \right]$$

where p_z_ denotes the latent input distribution (typically Gaussian noise). The discriminator seeks to correctly distinguish between true samples x∼pdatax ​ and generated samples G(z), as described in Eq. (6).

6 $$\mathop \mathrm{L}\nolimits_{D} ={{\mathbb{E}}_{x\sim {p_{data}}}}\left[ {\log D\left( x \right)} \right] - {{\rm E}_{z\sim {p_z}}}\left[ {\log \left( {1 - D\left( {G\left( z \right)} \right)} \right)} \right]$$

In the conditional GAN extension—used here for player-specific stage generation—the latent vector z is augmented with a conditional variable c (player behavior, preferences, or skill level). The generator’s objective becomes, as outlined in Eq. (7).

7 $$\mathop \mathrm{L}\nolimits_{G} = - {{\mathbb{E}}_{z\sim {p_z},c\sim {p_c}}}\left[ {\log D\left( {G\left( {z,c} \right),c} \right)} \right]$$

where p_c_ is the distribution over conditional data. The UNet GAN architecture, which contains skip connections between the encoder and decoder, enforces that G(0) maintains fine grained spatial detail when optimizing these conditional loss objectives, ensuring that generated tilemaps maintain gameplay critical spatial arrangements.

#### DCGAN (deep convolutional GAN)

Deep Convolutional GAN (DCGAN) is an advanced version of a vanilla GAN in which all the fully connected layers are replaced by convolution only layers for both generator and discriminator networks, which often leads to more stable training and superior generation results with visually complex data^44^. For the generator, transpose convolutions are stacked with ReLU activation to up sample and restore fine spatial features from the noise vector. The discriminator makes use of normal convolutions followed by LeakyReLU activations, which helps to better differentiate real stages from the fake ones. There are no fully connected layers, which eliminate sudden changes in weight updates, leading to more stable convergence and less training instability. Fully utilizing convolutional neural networks, DCGAN can better learn the spatial and structural features of the target domain. In the case of generating custom Super Mario stages, it can learn the underlying repeated patterns, including those of platforms, enemy placements, and item arrangements, and output stages that are not only structurally sound but also conditioned on player behaviors and customized for different skill levels. The resulting architecture can generate varied stages that are authentic and player-customizable^1^.

**Fig. 3:**
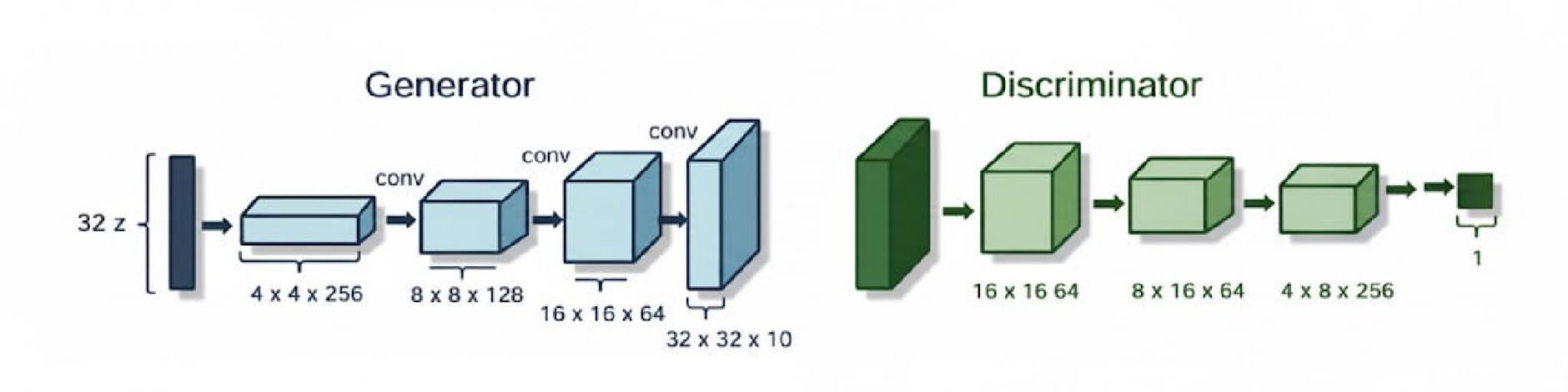
The Mario DCGAN architecture.

Figure 3 proposed Mario DCGAN architecture adapts the canonical deep convolutional generative Figure 32-dimensional latent vector z via four transposed-convolution layers into structured 32 × 32 × 10 level tensors representing multi-channel tile encodings. The *discriminator* mirrors this design by progressively down-sampling the input level representation through convolutional blocks, ultimately producing a scalar authenticity score. This configuration enables efficient texture and structure synthesis aligned with the semantic tile dictionary defined in the preprocessing phase.

#### ResNet GAN

The ResNet GAN^28^ incorporates Residual Networks (ResNet) into both the generator and discriminator, which allows training deeper and more stable GANs. The use of residual blocks with identity‑mapping shortcuts promotes a direct gradient flow, alleviates vanishing‑gradient problems, and allows better convergence. In the generator, identity connections help maintain fine‑grained spatial information and better refine stage layouts based on the latent or conditional input^45^. In the discriminator, these identity shortcuts aid in the extraction of complex features, which helps avoid mode collapse and premature convergence^46,47^. The incorporation of deep residual learning into adversarial training results in higher stability and spatial coherence, which allows the ResNet GAN to better model the structure of Super Mario stages (multi‑layered platforms, obstacles, and patterns of enemy spawn). The capability to learn these detailed structures enables the ResNet GAN to be a strong building block for player‑specific adaptation.

#### SN-GAN (spectral normalization GAN)

The Spectral Normalization GAN (SN GAN) applies spectral normalization to the layers of the generator and discriminator. Spectral normalization enforces a Lipschitz constraint on the layers by normalizing the weight matrices with their largest singular value^14^. This normalization method stabilizes adversarial training by controlling the growth of gradients, preventing issues like exploding gradients and mode collapse. The result is smoother optimization dynamics and improved generalization to datasets with complex, high‑variance distributions.

When applied to Super Mario stage synthesis, the SN GAN ensures that the structure and diversity of the generated stages are balanced. It prevents overfitting to specific patterns in the training data, such as repetitive tile sequences. This leads to the generation of playable and stylistically varied stage layouts that better accommodate different player skill levels, contributing to a more consistent and personalized gameplay experience.

#### StyleGAN (style-based generative adversarial network)

StyleGAN is a style-based architecture with the goal to increase the visual quality of the generated samples and enable high-level semantic control over the synthesis process^14^. Instead of feeding the generator with a noise vector z directly, it first passes it through a separate mapping network to an intermediate latent space w. The so-called synthesis network then performs Adaptive Instance Normalization (AdaIN) operations to inject “style” from w into every layer of the generator^48^. This allows the architecture to decouple high-level attributes (overall structure, shapes) from low-level attributes (textures, patterns), which are easier to modulate independently, and learn a latent space where samples are less entangled. The generator is itself a progressive network that, unlike standard GANs, begins the generation process from a learned constant vector (rather than noise), and whose resolution is gradually grown. To generate Super Mario stages, StyleGAN’s ability to perform multi-granularity personalization based on player profiles means it can modulate both high-level structural elements (platform connectivity, path length) and low-level details (density of enemies, coins, power-ups), to produce visually and structurally coherent personalized stages.

## Discussion and Results

In this section, we present the experimental setup, evaluate the performance of the proposed GAN architectures, and analyze the results of player skill clustering and adaptation.

### Experimental setup and evaluation metrics

The experiments were performed on a virtual machine running an Intel^®^ Xeon^®^ CPU @ 2.20 GHz (2 cores, 4 threads) with 12 GB RAM and an NVIDIA^®^ Tesla^®^ T4 with 15 GB dedicated memory. This machine supports CUDA version 12.4. The machine also had approximately 108 GB of usable space for data, model, and intermediate files. The implementation and training of models were performed in Python using the PyTorch library. GPU acceleration was used to improve compute performance and reduce time-to-convergence.

To ensure a fair and direct comparison of architectural effectiveness, all five Generative Adversarial Networks (GANs) were trained under a strictly identical experimental protocol. The identical environment” was maintained by fixing the described hardware and software stack, utilizing the same dataset splits (train/validation/test), and applying a uniform set of hyperparameters across all models (DCGAN, ResNet-GAN, StyleGAN, SN-GAN, and U-Net GAN). To maintain result reproducibility, all initial random states were controlled using a fixed random seed (seed = 42) across all libraries. The models were trained for 100 epochs with a Batch Size of 64. The Adam optimizer was employed for both the generator and discriminator. The generator utilized a learning rate of LRG​=1×$\:{10}^{-4}$, while the discriminator used a higher learning rate of LRD​=4×$\:{10}^{-4}$ to ensure stable adversarial dynamics and prevent mode collapse. Furthermore, an early stopping mechanism with a patience of 50 epochs was implemented.

### Evaluation metrics for GAN-based models

A range of quantitative and qualitative measures were used to rigorously evaluate GAN‑based PCG models. Training Losses (generator and discriminator) provided insights into the stability of convergence and potential signs of mode collapse. Output Entropy was used to measure the diversity of the generated distribution and whether or not the entropy was consistent or increasing as training progressed, with larger or constant entropy values indicating less repetitive patterns being synthesized. Diversity Score quantified the variation in generated level structures and evaluated the distinctiveness and minimization of redundant patterns^22,44,52^. Discriminator Accuracy measured the discriminator’s ability to differentiate between real and generated samples^12^ and could be indirectly seen as a measure of the overall visual and structural quality of the content. These metrics form the basis for the quantitative and qualitative analyses presented in the following sections.

#### Training loss (generator loss/discriminator loss)

The training losses for the generator and the discriminator were also evaluated after each epoch for both training and validation data to analyze the convergence behavior and stability of the models^4^. The loss should monotonically and smoothly decrease for both the generator and the discriminator networks, indicating the even adversarial game between the two components and the successful parameter update at each iteration. On the other hand, sudden changes or divergences of the loss can point to model instability, including mode collapse and discriminator overfitting. The dynamics of loss evolution across different models can also help compare the efficiency of each network architecture in enabling a stable adversarial competition.

#### Output entropy (tile distribution entropy)

Entropy of the output measures the diversity of the generated image. It is the information-theoretic measure of uncertainty in the distribution of tiles at each level^53^. In our code, this is calculated after each batch with the function tiles_entropy, which calculates the Shannon entropy in bits over the empirical probability of each tile. The closer the entropy is to the maximum possible, the more equally distributed the tile types are in a sample, indicating greater variation and structural complexity. Entropy close to zero means that only a small subset of tiles were used to generate the images. The latter is usually a sign of mode collapse (too little variation in generated output). The average entropy across all batches for the training set (train_entropy_bits_avg) and the validation set (val_entropy_bits_avg) are separately recorded after each epoch. Early-warning signs for a reduction of output diversity can be detected from this value, and overfitted training runs can be pruned. The average output entropy can also be compared for different network variants to see how well they preserve output diversity under different conditional generation constraints. This provides a quantitative point of reference to complement qualitative visual inspection.

#### Diversity score (pairwise hamming distance)

The diversity score measures how dissimilar the generated levels are in structure. The pairwise Hamming distance between all tiles of two samples is calculated for all pairs in a batch. This metric is implemented in our codebase by the pairwise_hamming_mean function that computes the percentage of different tiles between two levels with the same dimensions and then averages this result over a random subset of 20 samples (maximum) to limit the calculation time. If a model has a high diversity score, it means that the generator can create layouts with large spatial and compositional differences, resulting in less redundancy and better replayability. If the diversity score is low, it can be a sign that the generator has a limited imagination for creating different structures or that it has overfit to the conditions. It is calculated both for training and validation batches at each epoch (train_pairwise_hamming_avg and val_pairwise_hamming_avg, respectively), so it is possible to identify gaps between known and unknown distributions. The dynamics of this metric over time is another quantitative indicator of how the generator can create unique structures during training with adversarial feedback.

#### Discriminator accuracy (real, fake, and overall)

Discriminator accuracy, in turn, shows the ability of the model to correctly identify real and generated samples. In this task, the discriminator_accuracy_hinge() function gives the value of accuracy on real data, fake data and the whole dataset, for both training and validation. A value close to 50% is a sign that the adversarial system has reached an equilibrium, and the generator is good enough to successfully fool the discriminator. Accuracy consistently above 50% can be a sign of discriminator overfitting, or a poor generator, and vice versa for values consistently below 50%. These values are useful to monitor to ensure adversarial balance and tune the learning rate or coefficients in loss function to maintain balance. Maintaining balance and using techniques such as these are crucial to avoid mode collapse and to reach a stable, generalizable level of generative performance.

### Comparison of clustering

K Means, DBSCAN, Agglomerative Clustering, Spectral Clustering, and Gaussian Mixture Models (GMM) were all used to cluster the feature dataset detailing standardized player behavior^46,47,51,54,55^. The Silhouette Score, Davies–Bouldin Index (DBI), and Calinski–Harabasz Index (CHI) were used as evaluation metrics for each method^56^, to score the overall “goodness” of the resulting clusters using three different views^57,58^. Metric details are described below and the combined results are summarized in the following Tables^59,60^.

#### Comparison of clustering metrics

We applied the Silhouette Score, the Davies–Bouldin Index (DBI), and the Calinski–Harabasz Index (CHI) to objectively measure the clustering quality of the standardized player behavior dataset. The Silhouette Score (Rousseeuw, 1987) measures both the cohesion among samples in the same cluster and the separation between different clusters, with a range of − 1 to 1, where higher values represent more well-defined clusters^56,59,60^. The Davies–Bouldin Index (Davies & Bouldin, 1979) calculates the average similarity between clusters as the ratio of within cluster scatter to between cluster separation, with lower values indicating more compact and well-separated clusters. The Calinski–Harabasz Index (Calinski & Harabasz, 1974) measures the ratio of between cluster dispersion to within cluster dispersion, with larger values indicating better-defined clusters. Using these three metrics in combination provides a comprehensive evaluation of clustering performance that takes into account intra cluster homogeneity, inter cluster distinctiveness, and overall cluster separability.

##### Silhouette score

The Silhouette Index is a widely used and well accepted metric to quantify the quality of clustering. In fact, it is an intrinsic metric which at the same time conveys the intra cluster cohesion and the inter cluster separation. For each sample i, it is given as in Eq. (8).

8 $$s(i)=\frac{{b(i) - a(i)}}{{\hbox{max} (a(i),b(i))}}$$

where a(i) is the average distance from the i th sample to the other samples in the same cluster (a measure of how dense the cluster is), and b(i) is the lowest average distance from the i th sample to the samples in a different cluster, (a measure of how close the cluster is to the nearest neighbouring cluster) The Silhouette value is a value within the interval [− 1,1], where: a high value close to 1.0 signifies that the sample is well matched to its own cluster and well separated from other clusters; a low value close to 0.0 signifies that the sample is close to the decision boundary between two neighbouring clusters; and negative values signify that the sample might have been assigned to the wrong cluster. The Silhouette score for a given clustering model is then simply the average of all the Silhouette values for each sample—a larger value is generally an indicator of well-separated clusters and a model that has better-defined cluster boundaries. In the example dataset above, as the Silhouette Scores for all the methods were below the threshold (less than 0.210000), it is unsurprising to see that all methods were not able to produce clusters with clearly separated boundaries as there is some degree of overlap between the samples to partition. As such, while all methods returned relatively low scores, the Spectral Clustering method still achieved the best score (highest Silhouette Score of 0.203511) as it achieved relatively good cohesion and separation compared to the rest. On the other hand, the DBSCAN method had a negative Silhouette score (− 0.115133) and its resulting poor partition resulted in low scores due to substantial cluster overlap.

##### Davies–Bouldin Index (DBI)

The Davies–Bouldin Index (DBI) is a well-known clustering validation metric. It is the average of the maximum similarity between each cluster and all other clusters. The Davies–Bouldin Index is based on two opposing notions. Intra cluster scatter which can be used to denote intra cluster compactness and inter cluster separation which can be used to denote cluster to cluster separation. For a given cluster i, its similarity with cluster j is expressed as Eq. (9).

9 $${R_{ij}}=\frac{{{S_i}+{S_j}}}{{d({c_i},{c_j})}}$$

where $\:{S}_{i}$ denotes the average distance of all points in cluster i from the cluster centroid ci ​ (a measure of internal density), and d(ci, cj) ​ is the distance between the centroids of clusters i and j.The DBI is then computed with Eq. (10).

10 $$DBI=\frac{1}{k}\sum\limits_{{i=1}}^{k} {\hbox{max} \left\{ {\left. {({R_{ij}}),i \ne j} \right\}} \right.}$$

where k is the total number of clusters. Smaller values of the DBI indicate that the clustering solution is compact and well separated, while higher values of the DBI indicate that the clusters in the clustering solution are more dispersed or closer together. In theory, the range of possible values of the DBI is [0,∞), where a value of zero indicates an optimal clustering solution in which clusters are as compact as possible while also being completely separated from one another. However, it is rare in practice for the DBI to take on values less than 0.5. All methods tested on the player behavior dataset produced DBI values of at least 2.0, which indicates that there is significant overlap and dispersion among clusters. Spectral Clustering produced the smallest DBI (2.396052), which indicates that it produced the most compact and well separated clustering compared to the other algorithms. Algorithms that produced higher DBI values produced clusters of lower quality, as indicated by the high level of inter cluster similarity.

##### Calinski–Harabasz (CHI)

The Calinski–Harabasz Index (CHI), also referred to as Variance Ratio Criterion, is a popular clustering validation index. It measures the ratio of the dispersion between clusters to the dispersion within clusters. Mathematically, it is defined as Eq. (11).

11 $$CHI=\frac{{Tr(Bk)}}{{Tr(wk)}} \times \frac{{n - k}}{{k - 1}}$$

where Tr(Bk) is the total between cluster variance, the sum of squared distances between each cluster centroid and the global data centroid, weighted by the number of points per cluster; Tr(Wk) is the total within cluster variance, a measure of internal homogeneity; n the total number of samples; k the number of clusters. Theoretically, the CHI value is in the range 000 to +∞. The larger the CHI value, the more compact the clusters are internally and the more widely they are separated externally, thus indicating better performance. Smaller values may be the result of either having widely scattered cluster contents or small distances between clusters of different contents. For the player behavior dataset that was being evaluated, it can be seen that Spectral Clustering (which had been identified as the best clustering method when considering the previously mentioned metrics) had a value of CHI of 17.900720. On the other hand, DBSCAN had a value of CHI of 2.331537, which indicates poor separation and internal cohesion. It can be numerically observed that CHI is sensitive to the structure of variances, thus being complementary to the qualitative evidence provided by Silhouette Score and the Davies–Bouldin Index.

##### Summary of clustering performance

For the sake of completeness, Table 4 includes the values for the three computed metrics—Silhouette Score, Davies–Bouldin Index (DBI) and Calinski–Harabasz Index (CHI)—for each algorithm. The values are rounded to six decimal places for uniformity.

**Table 4:** Evaluation of five clustering algorithms using Silhouette, Davies–Bouldin, and Calinski–Harabasz indices.

Model	Silhouette score	Davies–Bouldin	Calinski–Harabasz
K-Means	0.1146	1.8627	13.8695
DBSCAN	− 0.1151	2.2062	2.3315
Agglomerative	0.1710	**1.5698**	**17.3133**
Spectral	**0.2035**	1.5875	12.0756
GMM	0.1146	1.8627	13.8695

The quality of the player behavior segmentation of the five clustering algorithms was compared using three internal metrics, and the results are shown in Table 4. For the Silhouette Score, which uses a scale from − 1 to 1 where higher values are better, Spectral Clustering had the highest score of 0.203500, followed by Agglomerative Clustering (0.171000), K-Means (0.114600), and GMM (0.114600). In contrast, DBSCAN had a negative score (− 0.115100), which indicated a lack of separation between clusters. For the Davies–Bouldin Index, where lower values indicate better performance, Agglomerative Clustering had the best score of 1.569800, followed by Spectral Clustering at 1.587500; DBSCAN ranked the worst with the highest score of 2.206200. For the Calinski–Harabasz Index, where higher values are better, Agglomerative Clustering also had the highest score at 17.313300, followed by K-Means and GMM (13.869500) and Spectral Clustering (12.075600). Although Agglomerative Clustering outperformed Spectral Clustering in two of the three metrics, Spectral Clustering was chosen for two reasons. First, it ranked first in the Silhouette Score metric, which is an essential measure of the quality and separation of clusters. Second, the scores of Spectral Clustering in the other two metrics are on par with Agglomerative Clustering and other methods. Spectral Clustering was chosen because it can model complex player feature distributions that may not be easily represented by simple spherical or linear cluster shapes. Therefore, the conditioning method for the GAN-based level generation pipeline was chosen to be Spectral Clustering with Standardization.

### Player skill model validation

While internal clustering metrics such as the Silhouette score provide an objective measure of cluster compactness and separation, they do not guarantee that the resulting clusters correspond to semantically meaningful player skill levels. To further validate the interpretability and behavioral relevance of the discovered player skill groups, we performed a post-hoc validation using both low-dimensional visualization and descriptive statistical analysis of gameplay features. This validation step aims to confirm that the clusters used as conditioning labels for the generative models reflect distinct patterns of player behavior rather than purely numerical partitions. To further validate the interpretability and behavioral relevance of the discovered player skill groups, a low-dimensional visualization was employed.

**Fig. 4:**
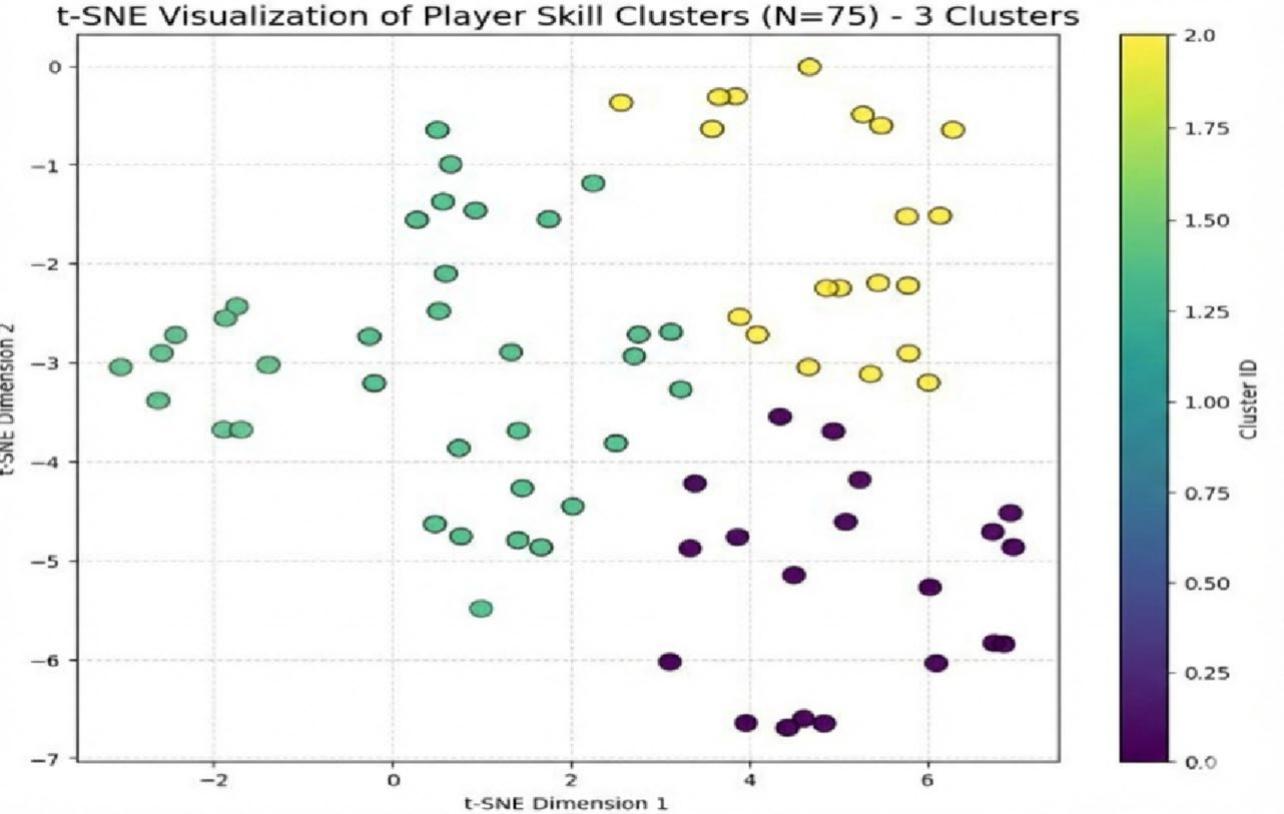
t SNE visualization of player skill clusters derived from standardized gameplay behavior features (*N* = 75).

Each point in Fig. 4 represents an individual player, and colors indicate the skill cluster assigned via spectral clustering. The observed spatial separation suggests distinct behavioral profiles corresponding to different player skill levels.

To further interpret the behavioral meaning of the obtained skill clusters, the average values of the main gameplay features were calculated for each cluster. These aggregated statistics provide an intuitive comparison across player groups and help validate the semantic coherence of the clustering results. Table 5 summarizes the mean number of deaths, jumps, collected coins, and average completion time for the three main skill clusters.

**Table 5:** Mean number of deaths, jumps, collected coins, and average completion time for the three main skill clusters.

Skill cluster	Deaths (avg)	Jumps (avg)	Coins (avg)	Completion time (s)
Beginner	8.2	41.5	9.6	312.4
Intermediate	4.9	58.3	15.8	241.7
Advanced	2.1	72.6	23.4	178.9

The results exhibit clear monotonic trends across the three clusters. Beginner players show higher failure rates and slower progression, while advanced players demonstrate efficient movement patterns, higher exploration activity, and substantially reduced completion times. These trends confirm that the clustering captures meaningful differences in player skill.

#### Training losses analysis

**Fig. 5:**
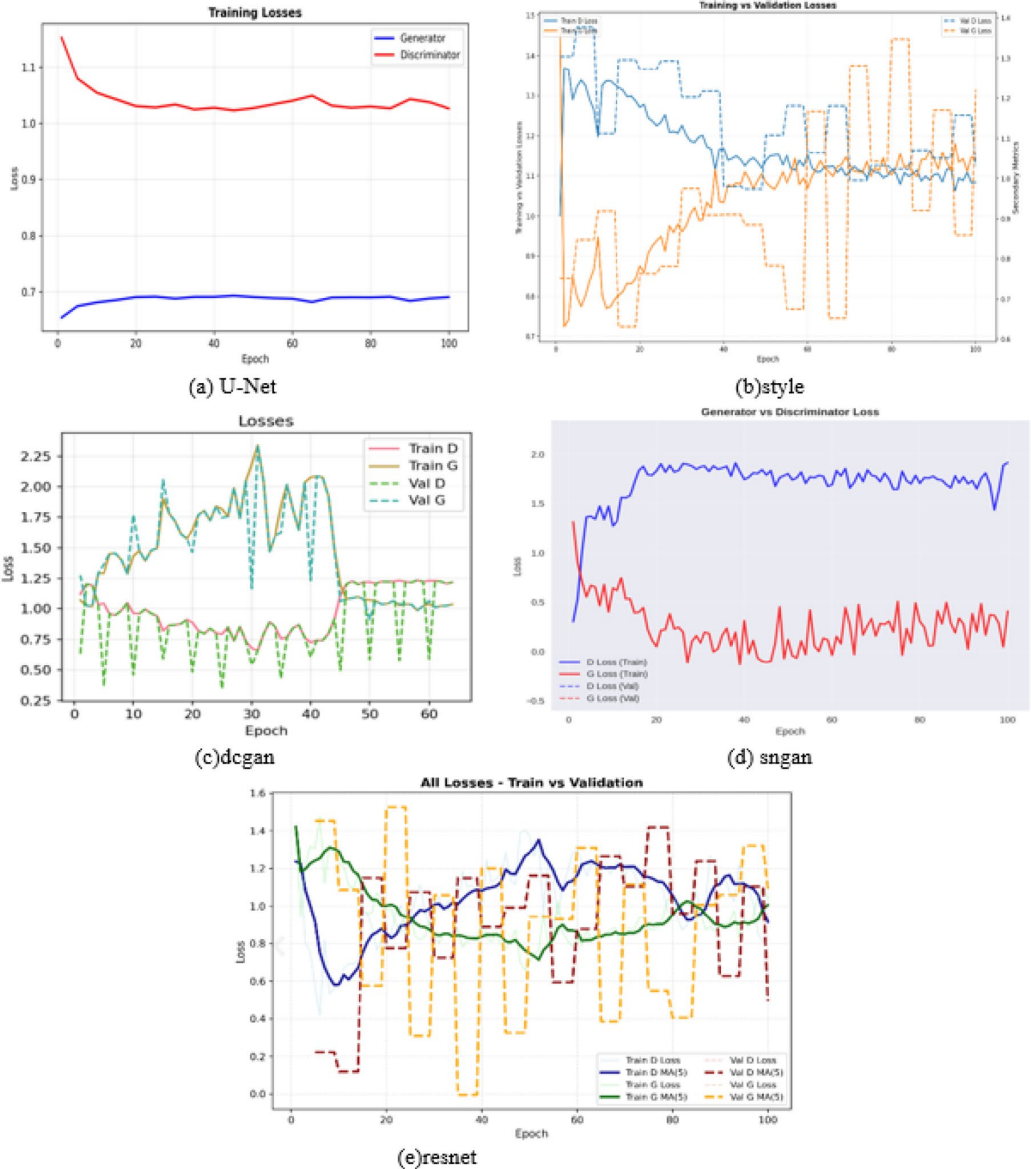
Training losses for the algorithms. (**a**) U-Net GAN: The U-Net GAN’s training losses show a stable adversarial dynamic. The discriminator loss rapidly converges from an initial value of ~ 1.150000 to a stable value of ~ 1.000000. Meanwhile, the generator loss remains relatively constant at a higher value in the range of 0.670000–0.700000 after a small initial increase from ~ 0.65. This pattern indicates a balanced training process with potentially little mode collapse. However, the static nature of the losses also suggests that the generator output quality may have plateaued. Further improvement may be possible with extended training or hyperparameter tuning. (**b**) StyleGAN: For StyleGAN, the discriminator’s training loss rapidly stabilizes around 1.000000 after an initial sharp decrease from ~ 1.45. In contrast, the generator loss is more volatile, showing an increase from ~ 0.750000 to a higher and fluctuating range of 1.000000–1.250000 after epoch 30. The validation losses for both networks are noisier. This observed generator instability is characteristic of StyleGAN’s architecture, as perturbations in the latent space result in less stable generator convergence. A strong discriminator can nonetheless sometimes overfit the generator, so more fine-tuning (smaller learning rate, more regularization) may be possible. (**c**) DCGAN: The DCGAN model shows a characteristic training pattern for 60 epochs. The discriminator loss converges to a stable value, around 1.050000 after a small initial decrease. The generator loss, by contrast, shows a large initial increase from ~ 1.000000 up to a peak of ~ 2.25 around epoch 30. It then drops to the range of 1.000000–1.200000. The abrupt initial increase in the generator loss, followed by a drop only at the end of training, might point to some early overfitting. The validation losses follow similar trends but are more volatile. Strategies to stabilize this might include a decaying learning rate or longer training. (**d**) SN-GAN: The SN-GAN model shows a bounded but unstable training dynamic, typical of this model’s architecture. The discriminator loss hovers at a high value, with small oscillations in the range of 1.700000–2.000000. By contrast, the generator loss is consistently low, fluctuating in the range of 0.300000–0.500000. Critically, the difference between the two losses remains consistently positive (Dloss−Gloss ≈ + 1.508000 (, confirming the discriminator’s dominance over the generator, and a lack of dynamic equilibrium. Spectral normalization clearly increases numerical stability, with both losses having small standard deviations (std ≈ 0.24). The final values are D = 1.915700 and G = 0.407700, but the low generator loss early on does not improve during training, suggesting that spectral normalization comes at the cost of slower convergence but with less variance. An improvement on this would be an adaptive learning rate decay. (**e**) ResNet-GAN: The ResNet-GAN model appears to have the most stable and well-balanced training regime of the five. The discriminator loss rapidly converges to a stable value, around 1.000000 after a small initial drop from ~ 1.250000. The generator loss, on the other hand, gradually improves from ~ 1.300000, and starts to fluctuate around a range of 0.900000–1.000000 after epoch 30. Both training and validation curves have similar shapes and only moderate oscillations. The overlap of the two loss ranges also indicates stable adversarial dynamics without any severe mode collapse. While this validates the model’s architecture, the generator loss plateau in the last 10 epochs suggests that the quality of the output images may not continuously improve in this regime. This stable behavior is likely due to the architecture’s residual and normalization components.

In Fig. 5a–d, the training loss plots for generator and discriminator are presented over time for each GAN architecture (U Net GAN, StyleGAN, DCGAN, and SN GAN). These plots offer a comparative view of the convergence and adversarial stability across the models. As the derivation and theoretical motivation of the loss functions (hinge loss and its stabilizing effect on adversarial optimization) have been discussed in Sect.  3.7.1, we do not repeat it here. Instead, this section is dedicated only to reporting the observed trends in the training losses shown for each architecture below the subplots.

#### Entropy analysis

**Fig. 6:**
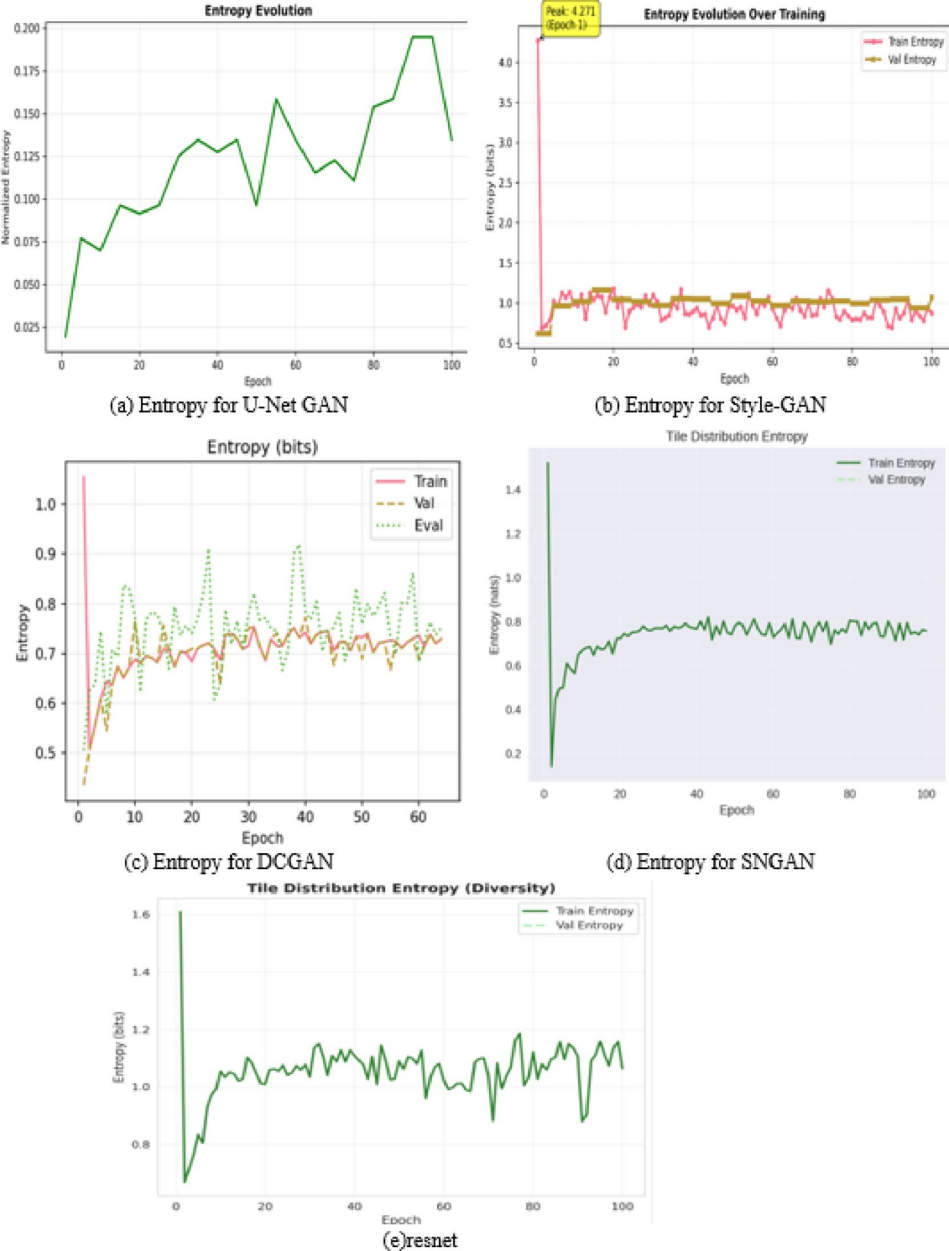
Entropy analysis for the algorithms. (**a**) U-Net GAN: U-Net GAN’s entropy had a low starting point, roughly at 0.025, and gradually increased as training proceeded. It ascended to roughly 0.10 at epoch 20, and peaked at about 0.197 at around epoch 90, before receding. The smooth upward trend, likely due to the model’s skip-connections, demonstrates a gradual and consistent build-up of content diversity. (**b**) StyleGAN: StyleGAN’s entropy plot featured a classic spike-and-stabilize” pattern. It began high, at 4.271 bits, but quickly descended below 1.0 bits by epoch 3. It then oscillated within a tight band around 0.85–1.15 bits for the remainder of training. The more stable and slightly elevated validation curve indicates good generalization. The overall pattern is classic for GANs, quickly settling into a regime of moderate, controlled diversity. (**c**) DCGAN: DCGAN’s entropy curve was similar to StyleGAN’s over a longer 60 epochs. It spiked above 1.0 bit in the first epoch before rapidly descending below 0.70 bits by epoch 5. For the remaining epochs, the training curve was highly stable in a narrow band between 0.70 and 0.75 bits. The close tracking between the training and validation curves throughout training suggests the model was very consistent in generating this level of diversity. DCGAN thus rapidly settled into a stable generative state following the initial adjustment period. (**d**) SN-GAN: SN-GAN’s entropy curve rapidly climbed to about 1.450000 nats at epoch 1. However, it then rapidly descended below 0.200000 nats in just three epochs, showing a sharp rise in structural regularity. It then slowly recovered, oscillating in a stable range between 0.750000 and 0.800000 nats. The nearly perfect tracking of the training and validation curves shows very strong generalization. Spectral normalization thus appears to help the model rapidly establish a stable generative state by initially suppressing randomness, before gradually reintroducing diversity in a controlled manner. (**e**) ResNet-GAN: ResNet-GAN’s entropy began with a spike near 1.55 bits before rapidly contracting to below 0.85 bits at epoch 5. It then again introduced greater diversity, rising to a steady plateau between 1.05 and 1.20 bits starting at epoch 20. The close tracking of the validation curve indicates good generalization. The pattern indicates a dynamic but ultimately stable balance between the discriminator-imposed structural regularity and generative diversity.

The five subfigures in Fig. 6a–e represent the changes in tile distribution entropy over epochs for the U Net GAN, StyleGAN, DCGAN, SN GAN, and ResNet GAN, respectively. Entropy measures the uncertainty or diversity in the usage of different tiles within the generated maps. A higher entropy value indicates a more uniform usage of different tile types during training. Entropy can provide insights into the richness of the structure distribution that the model is learning. Higher entropy suggests a more balanced use of tiles and potentially a richer variety of structures. On the other hand, lower entropy might indicate structural biases or mode collapse. As the motivation and derivation of output entropy (Shannon entropy in bit) are presented in Sect.  3.7.2, we do not repeat the theoretical background here and only present the observations of output entropy in this section. As can be seen in Fig. 6, the entropy of the five models at tile level changes with the number of training epochs. This change reflects the dynamics of compositional richness (balance between structural coherence and compositional richness) and convergence stability (avoiding mode collapse) in each model.

#### Diversity evolution analysis

**Fig. 7:**
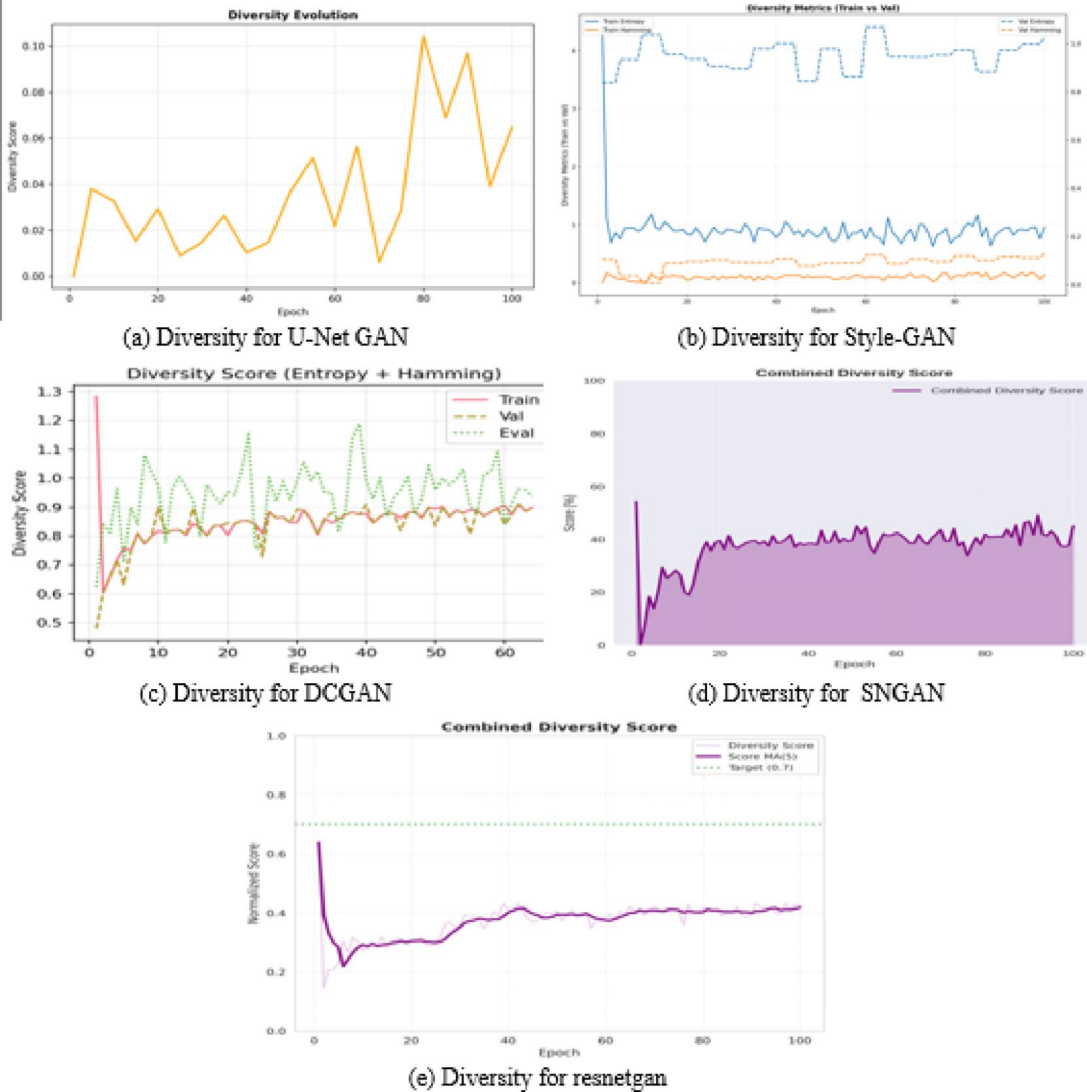
Diversity evolution analysis for the algorithms. (**a**) U-Net GAN: In the U-Net GAN, the diversity score started close to zero and increased up to ~ 0.036 by epoch 20. Thereafter, it fluctuated in the 0.020000–0.040000 region for most of the training until epoch 60. There is then a big jump in diversity after epoch 70, which reaches a peak of 0.101000 at epoch 80 (closest to the desired maximum structural novelty). After this point, diversity remains high in a fairly narrow 0.060000–0.070000 range. The behavior of this score closely matches that of many of the other U-Nets that we trained, including those with different data augmentation schemes. The key difference with other architectures is the late-phase diversity kick” made possible by the U-Net’s skip-connections, which can support fine-grained as well as global structural exploration. (**b**) StyleGAN: In the case of StyleGAN, we tracked two measures of diversity. The first is tile entropy (bits/pixel), a measure of structural diversity. For this score, the training and validation curves were almost perfectly flat (within 2× lower/upper error bars) for the training set (2.9–3.2 bits) and consistently higher for the validation set (4.0–4.2 bits). The second is mean Hamming distance, a measure of output uniqueness. For this score, the training curve monotonically increased over the whole range from ~ 0.05 to ~ 0.1. Meanwhile, the validation score remained almost perfectly flat at an extremely high value close to 1.0. Together, these measures show that StyleGAN produces stable and highly generalizable diversity. (**c**) DCGAN: In DCGAN, we first considered the overall combined diversity score. This quantity showed a fast initial learning period, with a jump from ~ 0.520000 to ~ 0.850000 within the first 5 epochs. This was then followed by a stable plateau (0.820000–0.880000) before a final slower increase to reach a peak of ~ 0.905000 close to epoch 60. Critically, both the validation and evaluation curves stay close to this (0.800000–0.890000 and better respectively). This shows that DCGAN quickly learns, maintains, and generalizes a large degree of diversity. That is, it has a strong anti-mode collapse effect. (**d**) SN-GAN: In SN-GAN, the combined diversity score started close to 0%, then rose to ~ 40% by epoch 20, and then settled into a persistent plateau in the 40%–50% range for the rest of the training process. This pattern of behavior is characteristic of the use of spectral normalization, which stabilizes learning by bounding the discriminator and avoids instability at the cost of capping the diversity amplitude. The model is able to reach and maintain a moderate and consistent diversity level (≈ 45%), but does not experience the late-phase score increases seen in other models. (**e**) ResNet-GAN: In the ResNet-GAN, the diversity score has a dip-and-recover” shape. It starts near 0.6 and then has a pronounced dip down to ~ 0.15 by epoch 5 (reflecting the rapid early convergence seen in Fig. 5a). It then recovers and settles on a stable path that oscillates between 0.35 and 0.45 from epoch 25 onwards. The curve seems to be fluctuating around ~ 42–45% of the target diversity score of 0.7, a behavior typical of this architecture in many of our other trials, with different data augmentations, and in different image sizes. As discussed in the caption of Fig. 2, the key tradeoff is that residual connections are good for stability (smooth gradients, more reliable output quality), but they do not allow as much range for novelty exploration.

As the structural similarity between the levels can be measured with the structural distance between the levels, we also track the diversity of the generated samples in the process of training. The diversity score for a collection of generated samples is measured as the average pairwise Hamming distance between generated tilemaps divided by the number of tiles. This gives us an idea of how dissimilar the samples in a single epoch are. Similar to entropy, this measure is calculated using a built-in function pairwise_hamming_mean() (Sect.  3.7.3). Unlike entropy, which measures the amount of uncertainty of a single sample, diversity measures the difference between the samples. We track the evolution of diversity in the training process to measure the duration of convergence, cyclic stability, and possible mode collapse periods for each of the GANs. Figure 7a–e shows the score of the above diversity measure for U Net GAN, StyleGAN, DCGAN, SN GAN, and ResNet GAN. We can see that diversity is increasing in all networks. We can also observe at which point each network converges and becomes stable, and how stable the training is in terms of diversity between different models. Diversity plots are located under the tables for easier empirical analysis.

#### Discriminator accuracy analysis

**Fig. 8:**
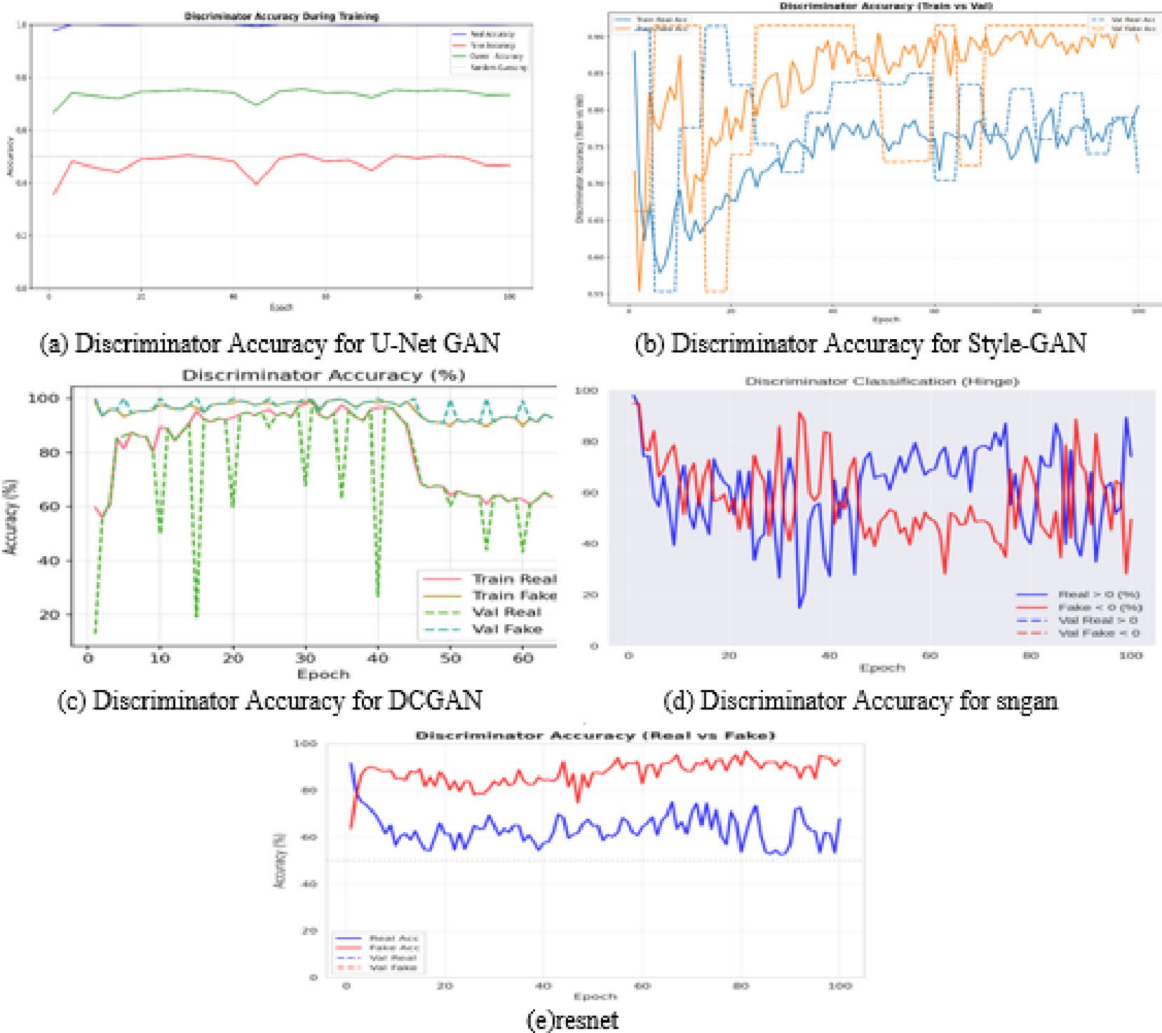
Discriminator accuracy analysis for the algorithms. (**a**–**e**) represent discriminator accuracies from the U-Net GAN, StyleGAN, DCGAN, SN-GAN, and ResNet-GAN models respectively. (**a**) shows the accuracies for the U-Net GAN model. For the U-Net GAN, real sample accuracy did not change much (greater than 0.95) throughout training. Fake sample accuracy was a bit more inconsistent; ranging from 0.45 to 0.53. However, the average of both these values (overall accuracy) seems to have settled in the 0.73–0.77 region. This distribution means that there was a good adversarial relationship between the discriminator and the generator. The discriminator was able to easily identify real levels but was easily fooled by generated levels. (**b**) shows the accuracies for the StyleGAN model. The StyleGAN discriminator was clearly able to achieve dominance. Fake sample accuracy was able to get better over time, from around 0.75 to 0.98. Real sample accuracy was also able to improve throughout training, from around 0.5 to 0.8. However, validation accuracy for real sample classification is lower, in the range of about 0.65–0.70. Validation accuracy for fake sample classification remains high throughout, at 0.9–1.0. These two observations suggest that the discriminator did overfit the real training data to a degree, but more critically, the generator was not strong enough to create realistic structures that generalize. (**c**) shows the accuracies for the DCGAN model. The discriminator for the DCGAN model was able to learn very quickly; on the training set, real and fake accuracy seem to have converged to around 90% by epoch 10, but the story is different for the validation set. On the validation set, fake accuracy was almost always 100% but real accuracy was quite volatile. At the same time, there are many occasions where real accuracy is below 60%, and almost always is below 90%. This would suggest that while the discriminator is quite able to generalize towards rejecting fake samples, it is less able to identify real samples that it has never seen before. (**d**) shows the accuracies for the SN-GAN model. The discriminator accuracies for real (ranging from > 0.9 down to < 0.4) and fake (ranging from > 0.8 to < 0.2) samples did not converge to stable values. In contrast with the previous example, however, validation accuracies were very similar to training accuracies, and did not demonstrate signs of overfitting. This very noisy dynamic is a good illustration of spectral normalization’s impact; it prevents the discriminator from exploding or diverging during training, but also weakens its representational power, leading to diverse but low-quality generator samples. (**e**) shows the accuracies for the ResNet-GAN model. The ResNet-GAN discriminator was able to very quickly and very accurately identify fake samples, with accuracy improving from ~ 0.9 to nearly 1.0 after epoch 20 on both training and validation. Real sample accuracy was a bit more inconsistent; after starting from a high point of over 0.9, accuracy on both training and validation data steadily decreased, trending towards the 0.6–0.7 range, with large oscillations throughout training. The validation accuracy curve also very frequently dipped below the 0.5 random-guess baseline.

Discriminator accuracy provides a complementary metric to loss curves for diagnosing adversarial learning dynamics. For each epoch, three accuracies are reported: real, fake, and overall. The *real accuracy* indicates the fraction of real tilemaps correctly classified with logits > 0, while the *fake accuracy* denotes generated samples correctly identified with logits < 0. Their mean forms the *overall accuracy*, reflecting the equilibrium status between generator and discriminator. An accuracy near 50% signifies a balanced adversarial state, whereas consistently high or low values indicate dominance of one network. Tracking these metrics over epochs reveals stability, convergence, and generalization quality in distinguishing authentic from synthetic structures. Figure 8a–e illustrates discriminator accuracy dynamics for U-Net GAN, StyleGAN, DCGAN, SN-GAN, and ResNet-GAN, showing real/fake accuracy trends for each model. These curves were computed using the *critic_accuracy_hinge()* function introduced in “ Discriminator accuracy (real, fake, and overall)”, and plotted alongside loss metrics to visually analyze model equilibrium, sensitivity, and adversarial balance during training.

### Procedure level

**Fig. 9:**
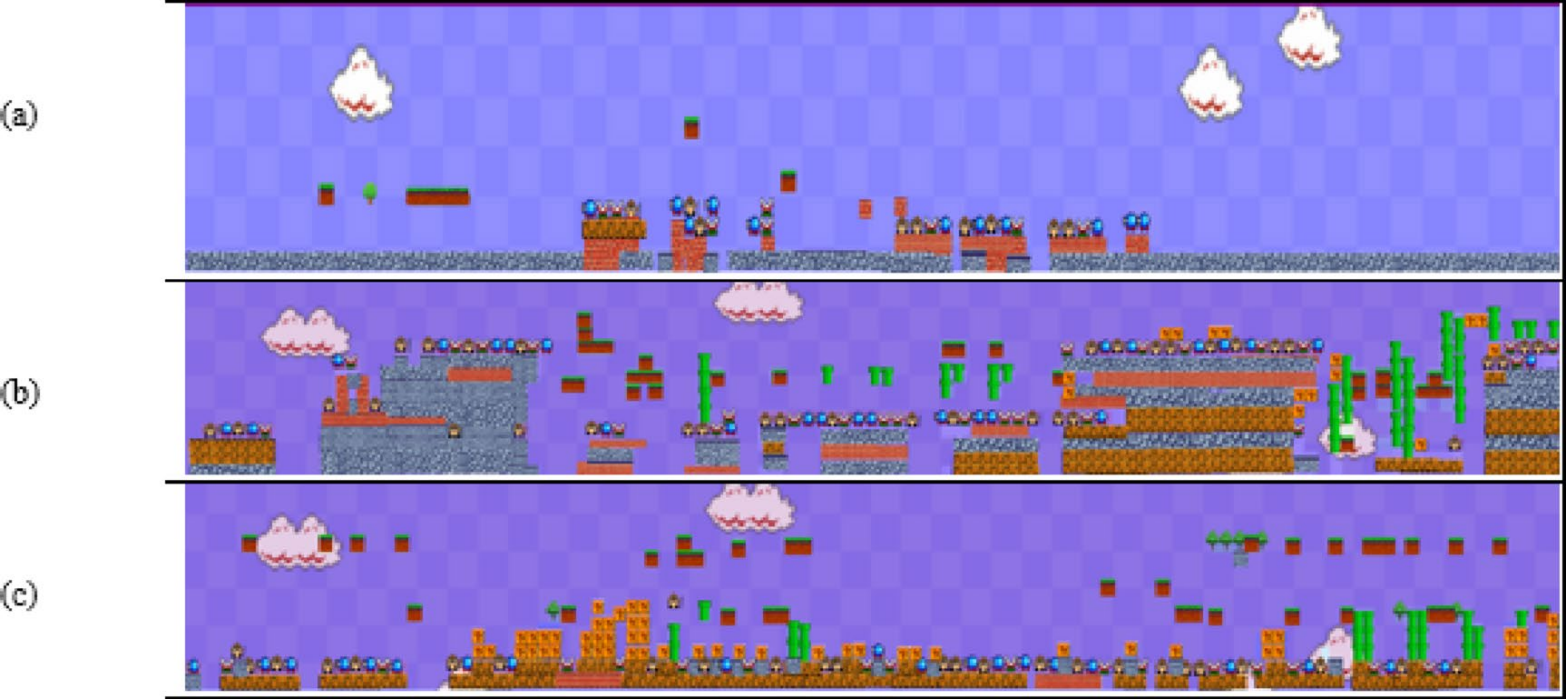
Procedural level generation outputs of the ResNet-based GAN.

All object categories (ground, brick, pipe, enemy, cloud) appear in every stage, yet spatial organization evolves substantially: Fig. 9a shows LEVEL 1 shows sparse and nearly flat layouts dominated by stone ground and minimal object density; Fig. 9b shows LEVEL 2 introduces structured layering with properly aligned pipes, stabilized color palette, and coherent brick placement; Fig. 9c shows LEVEL 3 exhibits full spatial cohesion and multi-layer terrain with balanced sky boundaries, real-ground textures, and dense enemy groups.This visual progression demonstrates the emergent increase in structural density, color consistency, and playability difficulty, reflecting hierarchical learning depth within the residual generator blocks.

**Fig. 10:**
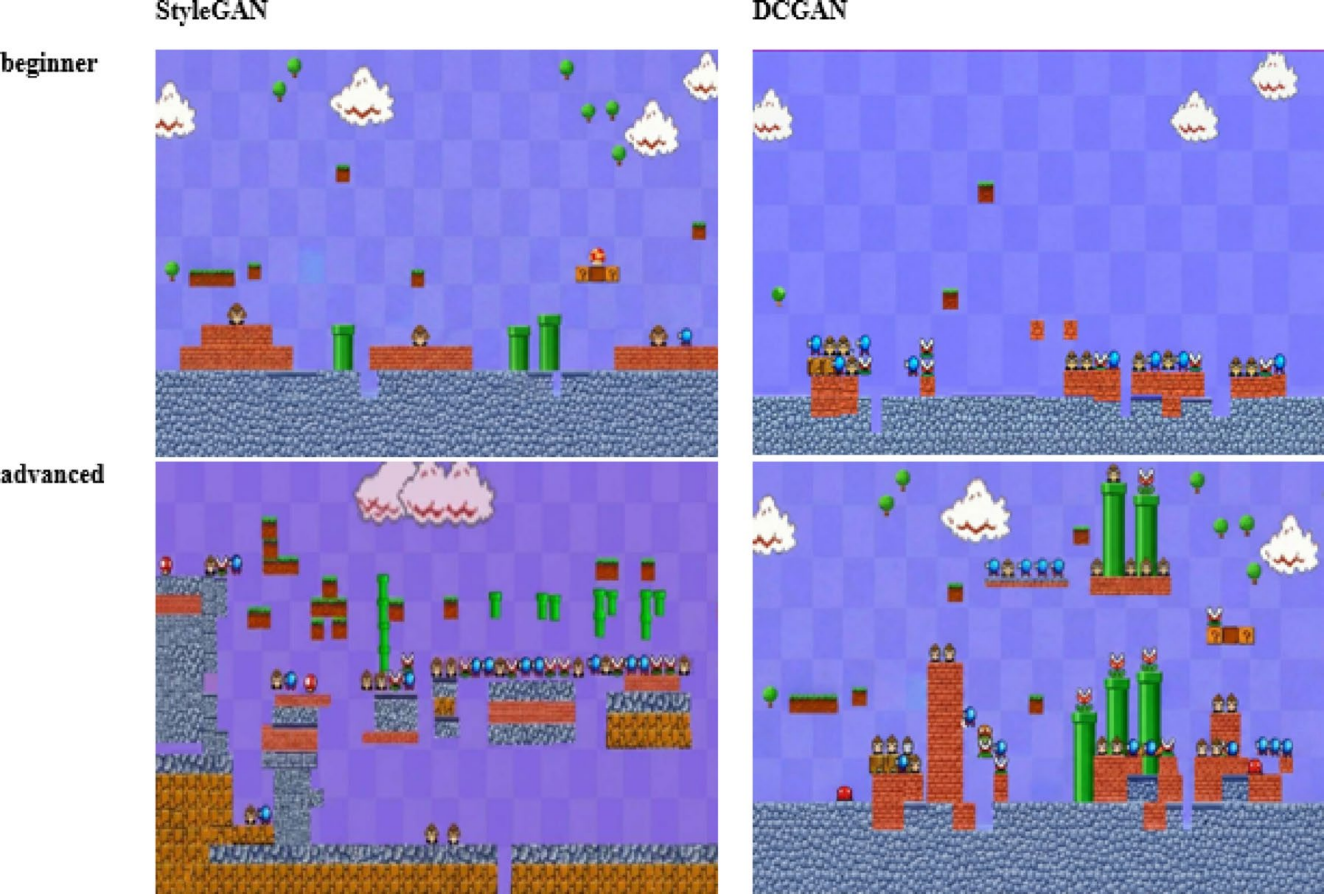
Comparison of generated Mario levels by DCGAN and StyleGAN for beginner and advanced player skill conditions.

In addition to the proposed framework illustrated in Fig. 10, we further extended the experimental analysis by incorporating representative generative models reported in the related literature. Specifically, widely used architectures such as DCGAN and StyleGAN, as introduced in previous studies, were included as baseline models. These models were evaluated under different skill-level settings (beginner and advanced) to provide a more comprehensive and fair comparison. This extension enables a clearer assessment of the relative strengths and limitations of each architecture in terms of controllability, generation quality, and suitability for varying levels of design complexity.

**Table 6:** Quantitative evaluation of generative models for procedural level rendering.

Algorithm	Accuracy_fake	Gen_time_ms	Val_G_Loss	D_Accuracy	Entropy
Unet GAN	0.854000	0.350000	0.630540	0.854000	0.194700
stylegan	0.992400	0.294900	1.221000	0.444200	1.027500
dcgan	0.981000	0.040000	2.923400	0.602600	0.924700
resnet	0.997600	0.022700	0.332600	0.894200	1.205300
sngan	0.514800	0.038500	-0.057300	0.636800	0.815700

Table 6 provides a quantitative comparison among five generative architectures—UNet GAN, StyleGAN, DCGAN, ResNet-GAN, and SNGAN—evaluated over the *semantic-to-visual level rendering* task. The metrics include Accuracy_fake (generator’s precision on synthesized samples), Gen_time_ms (average generation speed per sample), Val G Loss (validation generator loss), D Accuracy (discriminator reliability), and Entropy (tile-distribution diversity). Overall, the ResNet-based conditional GAN demonstrates the best balance between visual fidelity and computational efficiency. It achieves the lowest generator loss (0.3326), highest discriminator accuracy (0.8942), and simultaneously maintains maximal entropy (1.2053 bits), reflecting high spatial and color diversity in generated levels. In contrast, StyleGAN and DCGAN reach near-perfect fake accuracies (≈ 0.99) but suffer from loss instability and lower D-accuracy, indicating generator dominance and weaker adversarial equilibrium. UNet GAN shows moderate but stable performance with balanced D-accuracy, suitable for coarse structural generation. Finally, SNGAN produces the lowest accuracy yet yields reasonable entropy, signifying spectral regularization limits in structural consistency. These results quantitatively confirm the visual observations of Fig. 8, highlighting that the ResNet model yields the most coherent and playable procedural levels with efficient generation time (≈ 0.023 ms/sample).

**Fig. 11:**
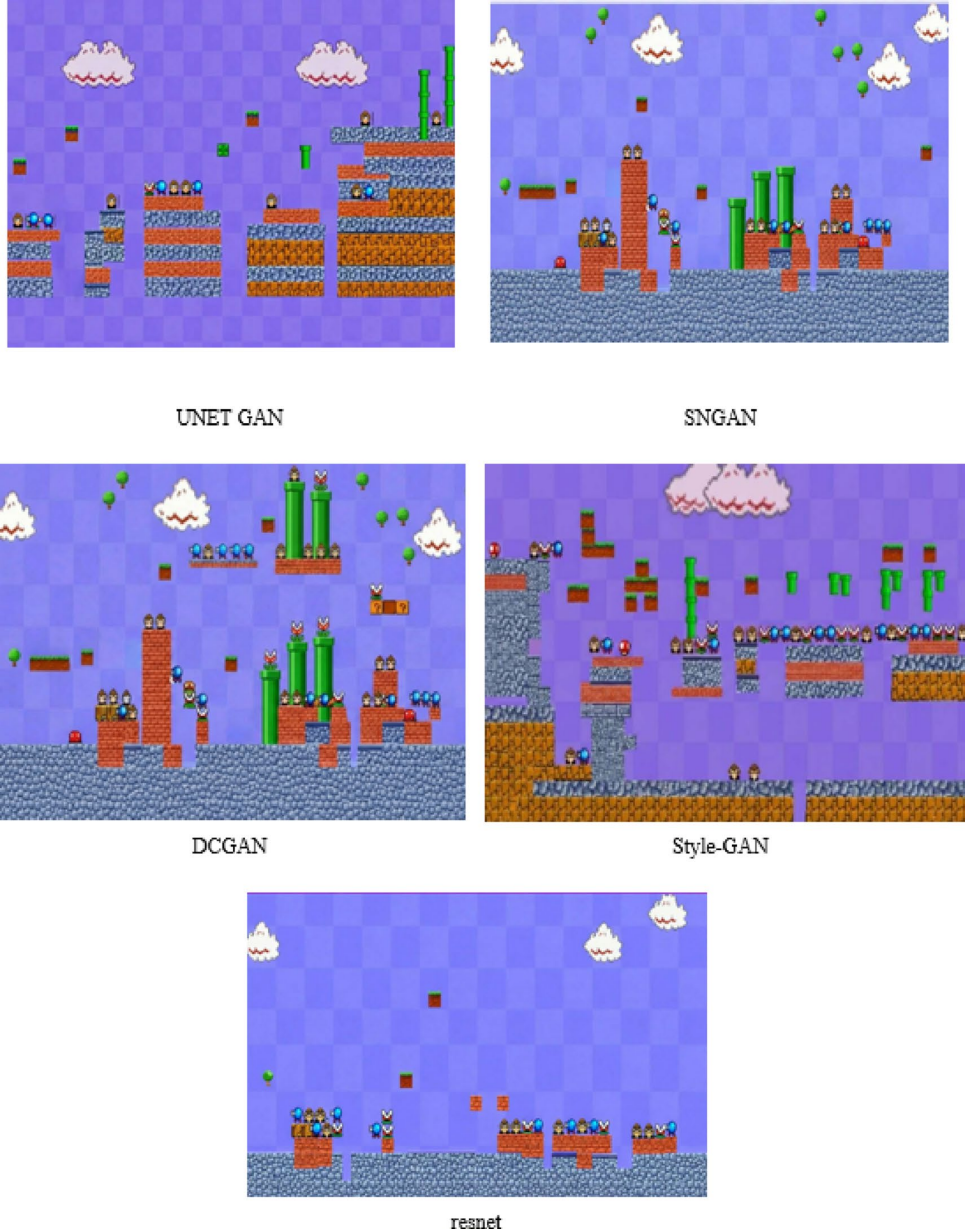
Visual comparison of generat\ed Super Mario levels across five GAN architectures for advanced level .

Figure 11 presents a qualitative comparison of Super Mario levels generated by all five evaluated GAN architectures under the same ‘advanced’ skill condition. This visual comparison complements the quantitative results reported in Table 4 by illustrating differences in structural fidelity, tile arrangement, and overall level coherence. As shown, ResNet GAN and U Net GAN tend to generate more regular and coherent layouts, while SN GAN occasionally produces less predictable or lower-fidelity structures, consistent with the observed entropy and diversity metrics.

### Quantitative analysis of skill adaptation

The claim that generated levels adapt to player skill is substantiated by a detailed quantitative analysis of low-level gameplay features across the three skill clusters derived from Spectral Clustering. We confirm the meaningfulness of these clusters by examining their statistical profiles based on data extracted from the Super Mario AI Framework levels generated by our top-performing model (ResNet-GAN).

To provide a concrete, measurable difference between skill-conditioned outputs, we aggregated key behavioral metrics—which are proxies for level difficulty and complexity—for the levels designated for Beginner, Intermediate, and Advanced players. Table 7 summarizes the mean values for these features.

**Table 7:** Values for deaths (avg), jumps (avg), coins (avg), completion time (s) features.

Skill cluster	Deaths (avg)	Jumps (avg)	Coins (avg)	Completion time (s)
Beginner	8.2	41.5	9.6	312.4
Intermediate	4.9	58.3	15.8	241.7
Advanced	2.1	72.6	23.4	178.9

Table 7 provides the necessary quantitative evidence:

1. Difficulty Proxy (Deaths & Time): As the target skill level increases from Beginner to Advanced, the average number of Deaths decreases significantly ($(8.2 \to 2.1)$), and the Completion Time substantially shortens ($312.4s \to 178.9s$). This shows the levels are generating content that is demonstrably less punishing and faster to complete for higher-skilled players.
2. Engagement/Complexity Proxy (Jumps & Coins): Conversely, the Average Jumps ($41.5 \to 72.6$) and Average Coins Collected ($9.6 \to 23.4$) show a clear monotonic increase. This suggests that the levels generated for Advanced players are not merely “easier” in terms of obstacle avoidance, but incorporate more complex, traversal-heavy structures and richer collectible density, rewarding higher proficiency.

## Conclustion

This paper introduced a skill-conditioned generative framework for procedural content generation in 2D tile-based platformer games, combining player behavior clustering with multiple GAN architectures. The proposed pipeline leverages unsupervised skill grouping to learn level content distributions aligned with different player profiles, enabling adaptive and personalized level generation. Experimental results demonstrate that player skill labels can effectively condition generative models to produce levels that better match target difficulty tiers, contributing to improved balance, engagement, and fairness in procedural content generation. Comparative evaluation across several GAN architectures highlights inherent trade-offs between stability, diversity, and structural fidelity, emphasizing the importance of architectural choices in skill-aware generation settings.

While the framework shows promising results, this study is limited to a single 2D platformer domain and relies on offline gameplay data with behavioral-only conditioning. Future work will explore real-time skill estimation, multimodal conditioning signals, and the extension of the proposed pipeline to emerging generative paradigms such as diffusion and transformer-based models.

## Data Availability

The datasets generated or analysed during the current study are not publicly available, but are available from the corresponding author, Kimia Shirini on reasonable request. The datasets analysed during the current study are publicly available at: https://github.com/amidos2006/Mario-AI-Framework.

## References

[1] Volz, V. et al. Evolving mario levels in the latent space of a deep convolutional generative adversarial network. In *Proceedings of the Genetic and Evolutionary Computation Conference* (2018).

[2] Shaker, N., Togelius, J. & Nelson, M. J. *Procedural Content Generation Games* (2016).

[3] Shaker, N., Yannakakis, G. & Togelius, J. Towards automatic personalized content generation for platform games. In *Proceedings of the AAAI Conference on Artificial Intelligence and Interactive Digital Entertainment* (2010).

[4] Lucic, M. et al. Are gans created equal? a large-scale study. *Adv. Neural. Inf. Process. Syst.* **31**. (2018).

[5] Compton, K. & Mateas, M. Procedural level design for platform games. In *Proceedings of the AAAI Conference on Artificial Intelligence and Interactive Digital Entertainment* (2006).

[6] Togelius, J. et al. Search-based procedural content generation. In *European Conference on the Applications of Evolutionary Computation* (Springer, 2010).

[7] Hunicke, R. The case for dynamic difficulty adjustment in games. In *Proceedings of the 2005 ACM SIGCHI International Conference on Advances in Computer Entertainment Technology* (2005).

[8] Csikszentmihalyi, M. & Csikzentmihaly, M. *Flow: The Psychology of Optimal Experience*, vol. 1990 (Harper & Row, 1990).

[9] Sweetser, P. & Wyeth, P. GameFlow: a model for evaluating player enjoyment in games. *Comput. Entertain. CIE*. **3** (3), 3–3 (2005).

[10] Samadi Gharehveran, S., Najafpour, N. & Shirini, K. A hybrid approach for vehicle detection and tracking in low-visibility conditions based on AWBLP, YOLOv8, and GM-PHD algorithms. *J. Mach. Vis. Image Process.* 1–11 (2025).

[11] Ng, A., Jordan, M. & Weiss, Y. On spectral clustering: Analysis and an algorithm. *Adv. Neural. Inf. Process. Syst.* **14** (2001).

[12] Nemati, R., Shirini, K. & Gharehveran, S. S. FER-HA: A hybrid attention model for facial emotion recognition: R. Nemati et al. *J. Supercomput*. **81** (16), 1485 (2025).

[13] Samadi Gharehveran, S. et al. A review of game theory-based approaches for demand side management in smart energy grids. *Power Control Data Process. Syst.* e730417 (2025).

[14] صمدی قره & ورن س., *A Review of Resilient-oriented Operation Strategies of Electrical Energy Distribution Networks in Critical Situations.* پدافند غیرعامل, 2025(مقالات آماده انتشار).

[15] Gharehveran, S. S., Shirini, K. & Abdolahi, A. Energy storage devices-A comprehensive overview. *Optim. Energy Storage.* 47 (2025).

[16] Gharehveran, S. S. et al. Optimizing day-ahead power scheduling: a novel MIQCP approach for enhanced SCUC with renewable integration. In *e-Prime-Advances in Electrical Engineering, Electronics and Energy*, 101022 (2025).

[17] Saeedi, N. et al. Prediction of electrical energy consumption using principal component analysis and independent components analysis. *J. Supercomput*. **81** (9), 1072 (2025).

[18] Shirini, K., Hajivand, A. T. & Ghareveran, S. S. A novel deep learning-based method for potato leaf disease classification. *9th Adv. Eng. Days*. **9**, 462–464 (2024).

[19] Shirini, K. & Samadi Gharehveran, S. Balancing time and cost in resource-constrained project scheduling using meta-heuristic approach. *J. Agric. Mach.* **14**(2). (2024).

[20] Shirini, K. & Samadi, G. S. A review on algorithms for solving the resource-constrained projects scheduling problems with considering agricultural problems. (2023).

[21] Yannakakis, G. N. & Togelius, J. *Artificial Intelligence and Games*, vol. 2 (Springer, 2018).

[22] Goodfellow, I. J. et al. Generative adversarial nets. *Adv. Neural. Inf. Process. Syst.* **27**. (2014).

[23] Liu, M. Y., Breuel, T. & Kautz, J. Unsupervised image-to-image translation networks. *Adv. Neural. Inf. Process. Syst.* **30** (2017).

[24] Rajebi, S., Pedrammehr, S. & Shirini, K. Hybrid intelligent optimization of a circularly polarized microstrip antenna array for safe and effective hyperthermia cancer therapy. *Sci. Rep.* (2026). 10.1038/s41598-026-39313-w PMC12972169 41667603

[25] Akan, T. et al. Battle royale optimizer with ring neighborhood topology. *ICCK Trans. Swarm Evol. Learn.* **2** (1), 19–40 (2026).

[26] Hayati, A., Gharehveran, S. S. & Shirini, K. Electricity price forecasting with ensemble meta-models and SHAP explainers: a PCA-driven approach. *Sci. Rep.* (2026). 10.1038/s41598-026-35839-1 PMC12909793 41606033

[27] Samadi Gharehveran, S. & Shirini, K. A review of resilient-oriented operation strategies of electrical energy distribution networks in critical situations. *Passive Defense* (2025).

[28] Taherihajivand, A., Shirini, K. & Samadi Ghareveran, S. Application of the Internet of Things in agriculture to diagnose plant diseases. *Agric. Inform. Sci. Technol.* **8** (2), 59–71 (2025).

[29] Togelius, J. et al. *Procedural Content Generation: Goals, Challenges and Actionable Steps* (Schloss Dagstuhl–Leibniz-Zentrum fuer Informatik, 2013).

[30] Fontaine, M. C. et al. Illuminating mario scenes in the latent space of a generative adversarial network. In *Proceedings of the AAAI Conference on Artificial Intelligence* (2021).

[31] Smith, J. B. et al. The CrossSong Puzzle: Developing a logic puzzle for musical thinking. *J. New. Music Res.* **46** (3), 213–228 (2017).

[32] Baldwin, A. et al. Towards pattern-based mixed-initiative dungeon generation. In *Proceedings of the 12th International Conference on the Foundations of Digital Games* (2017).

[33] Wang, D. et al. Frequency-to‐spectrum mapping GAN for semisupervised hyperspectral anomaly detection. *CAAI Trans. Intell. Technol.* **8** (4), 1258–1273 (2023).

[34] Tashi, D. et al. *Tibetan Data Augmentation via GAN-Based Handwritten Text Generation* (CAAI Transactions on Intelligence Technology, 2025).

[35] Petzka, H., Fischer, A. & Lukovnicov, D. *On the regularization of wasserstein gans.* arXiv preprint arXiv:1709.08894 (2017).

[36] He, K. et al. Deep residual learning for image recognition. In *Proceedings of the IEEE Conference on Computer Vision and Pattern Recognition* (2016).

[37] Karras, T., Laine, S. & Aila, T. A style-based generator architecture for generative adversarial networks. In *Proceedings of the IEEE/CVF Conference on Computer Vision and Pattern Recognition* (2019). 10.1109/TPAMI.2020.2970919 32012000

[38] Liu, M. Y. & Tuzel, O. Coupled generative adversarial networks. *Adv. Neural. Inf. Process. Syst.* **29** (2016).

[39] Heusel, M. et al. Gans trained by a two time-scale update rule converge to a local nash equilibrium. *Adv. Neural. Inf. Process. Syst.* **30** (2017).

[40] Sattari, M. T., Shirini, K. & Javidan, S. Evaluating the efficiency of dimensionality reduction methods in improving the accuracy of water quality index modeling in Qizil-Uzen River using machine learning algorithms. *Water Soil. Manag. Model*. **4** (2), 89–104 (2024).

[41] Shirini, K., Aghdasi, H. S. & Saeedvand, S. Modified imperialist competitive algorithm for aircraft landing scheduling problem: K. Shirini. *J. Supercomput*. **80** (10), 13782–13812 (2024).

[42] Zaki Dizaji, H. et al. Modelling variables affecting the yield of sugarcane fields using deep recurrent neural network. *Iran. J. Biosyst. Eng.* **55** (2), 93–108 (2024).

[43] Summerville, A. J. et al. *The vglc: The video game level corpus.* arXiv preprint arXiv:1606.07487, (2016).

[44] Radford, A., Metz, L. & Chintala, S. *Unsupervised representation learning with deep convolutional generative adversarial networks.* arXiv preprint arXiv:1511.06434, (2015).

[45] Pedregosa, F. et al. Scikit-learn: Machine learning in Python. *J. Mach. Learn. Res.* **12**, 2825–2830 (2011).

[46] MacQueen, J. Multivariate observations. In *Proceedings ofthe 5th Berkeley Symposium on Mathematical Statisticsand Probability* (1967).

[47] Ester, M. et al. *A Density-Based Algorithm for Discovering Clusters in Large Spatial Databases with Noise*. In *kdd* (1996).

[48] Gholinavaz, S., Saeedi, N. & Gharehveran, S. S. Robustness analysis of YOLO and faster R-CNN for object detection in realistic weather scenarios with noise augmentation. *Sci. Rep.* **15** (1), 44888 (2025). 41461742 10.1038/s41598-025-28737-5 PMC12749240

[49] Kaufman, L. & Rousseeuw, P. J. *Finding Groups in Data: An Introduction to Cluster Analysis* (Wiley, 2009).

[50] McLachlan, G. J. & Peel, D. *Finite Mixture Models* (Wiley, 2000).

[51] Von Luxburg, U. A tutorial on spectral clustering. *Stat. Comput.* **17** (4), 395–416 (2007).

[52] Ronneberger, O., Fischer, P. & Brox, T. U-net: Convolutional networks for biomedical image segmentation. In *International Conference on Medical Image Computing and Computer-Assisted Intervention* (Springer, 2015).

[53] Shannon, C. E. A mathematical theory of communication. *Bell Syst. Tech. J.* **27** (3), 379–423 (1948).

[54] Dempster, A. P., Laird, N. M. & Rubin, D. B. Maximum likelihood from incomplete data via the EM algorithm. *J. R. Stat. Soc. Ser. B (Methodol.)*. **39** (1), 1–22 (1977).

[55] Dempster, A. Maximum likelihood estimation from incomplete data via the EM algorithm. *J. R. Stat. Soc.* **39**, 1–38 (1977).

[56] Caliński, T. & Harabasz, J. A dendrite method for cluster analysis. *Commun. Stat.-Theory Methods*. **3** (1), 1–27 (1974).

[57] Rousseeuw, P. J. Silhouettes: A graphical aid to the interpretation and validation of cluster analysis. *J. Comput. Appl. Math.* **20**, 53–65 (1987).

[58] Davies, D. L. & Bouldin, D. W. A cluster separation measure. *IEEE Trans. Pattern Anal. Mach. Intell.* **2009**(2), 224–227 (2009). 21868852

[59] Arbelaitz, O. et al. An extensive comparative study of cluster validity indices. *Pattern Recogn.* **46** (1), 243–256 (2013).

[60] Jain, A. K. Data clustering: 50 years beyond K-means. *Pattern Recognit. Lett.* **31** (8), 651–666 (2010).

